# Gut microbiota is associated with the effect of photoperiod on seasonal breeding in male Brandt’s voles (*Lasiopodomys brandtii*)

**DOI:** 10.1186/s40168-022-01381-1

**Published:** 2022-11-15

**Authors:** Hanyi Zhu, Guoliang Li, Jing Liu, Xiaoming Xu, Zhibin Zhang

**Affiliations:** 1 State Key Laboratory of Integrated Management of Pest Insects and Rodents, Institute of Zoology, Chinese Academy of Sciences, Beijing, 100101 China; 2 University of Chinese Academy of Sciences, Beijing, 100049 China; 3 CAS Center for Excellence in Biotic Interactions, University of Chinese Academy of Sciences, Beijing, 100049 China

**Keywords:** Seasonal breeding, Photoperiod, Gut microbiota, HPG axis, Melatonin, Kisspeptin/GPR54 system

## Abstract

**Background:**

Seasonal breeding in mammals has been widely recognized to be regulated by photoperiod, but the association of gut microbiota with photoperiodic regulation of seasonal breeding has never been investigated.

**Results:**

In this study, we investigated the association of gut microbiota with photoperiod-induced reproduction in male Brandt’s voles (*Lasiopodomys brandtii*) through a long-day and short-day photoperiod manipulation experiment and fecal microbiota transplantation (FMT) experiment. We found photoperiod significantly altered reproductive hormone and gene expression levels, and gut microbiota of voles. Specific gut microbes were significantly associated with the reproductive hormones and genes of voles during photoperiod acclimation. Transplantation of gut microbes into recipient voles induced similar changes in three hormones (melatonin, follicle-stimulating hormone, and luteinizing hormone) and three genes (hypothalamic *Kiss-1*, testicular *Dio3*, and *Dio2/Dio3* ratio) to those in long-day and short-day photoperiod donor voles and altered circadian rhythm peaks of recipient voles.

**Conclusions:**

Our study firstly revealed the association of gut microbiota with photoperiodic regulation of seasonal breeding through the HPG axis, melatonin, and Kisspeptin/GPR54 system. Our results may have significant implications for pest control, livestock animal breeding, and human health management.

Video Abstract

**Supplementary Information:**

The online version contains supplementary material available at 10.1186/s40168-022-01381-1.

## Introduction

Seasonal breeding is a well-known phenomenon wherein organisms adapt to fluctuations in environmental conditions to improve the survival of offspring [1]. Changes in photoperiods have been widely recognized as a critical factor in controlling the seasonal breeding of animals [2], primarily through the hypothalamic-pituitary-gonad (HPG) axis [3]. Melatonin (MT) secreted by the pineal gland at night [4] regulates the release of hypothalamic gonadotropin-releasing hormone (GnRH) [5], which in turn induces secretion of luteinizing hormone (LH) and follicle-stimulating hormone (FSH) from the anterior pituitary [6], thereby stimulating growth of gonadal steroids (such as testosterone; T) secretion. In addition, several genes have been identified to regulate seasonal breeding in mammals through association with the HPG axis, such as iodothyronine deiodinase 2 (*Dio2*), iodothyronine deiodinase 3 (*Dio3*), Kisspeptin-1 (*Kiss-1*), G-protein-coupled receptor 54 (*GPR54*), a gene which encodes gonadotropin-releasing hormone (*GnRH*), and RFamide-related peptide 3 (*Rfrp-3*) [7–10]. The gene stimulated by retinoic acid 8 (*Stra8*), mainly expressed in spermatogonia, is necessary for entering meiosis [11], and its expression level reflects the degree of testis development [12]. Moreover, photoperiod also plays a vital role in regulating aggressive behavior in rodents. Classical neuroendocrine studies have suggested that testosterone and estradiol can directly regulate aggressive behavior in rodents [13], especially under short photoperiod [14].

Gut microbiota is composed of bacteria, fungi, protozoa, and archaea in the gastrointestinal tract, and their interactions with the host have been extensively investigated [15]. Gut microbiota is involved in many behavioral and physiological processes of the host [16, 17], including aggressive behavior [14], the secretion of reproductive hormones (such as T and MT) [18–20], spermatogenesis [21], and physiological metabolism [22, 23]. Dysbiosis of the microbial communities has been associated with male infertility in humans [24]. Variation in gut microbiota can be affected by both the host's internal factors (e.g., reproductive hormones and sex) [18, 25, 26] and external factors (e.g., diet and housing density) [27–29]. A few previous studies have suggested that photoperiod could change the composition of gut microbes in small rodents [14, 30]. Ren et al. (2020) found that the relative abundance of *Ruminococcaceae* is increased in Siberian hamsters (*Phodopus sungorus*) housed in short-day photoperiod condition [14]. Shor et al. (2020) found that long-day photoperiod significantly increases the relative abundance of *Lachnospira* but decreases the relative abundance of *Sharpea* in Siberian hamsters, as compared to short-day photoperiod [30]. Photoperiod can regulate the secretion of melatonin [31, 32]. Melatonin supplementation can increase the relative abundance of *Lactobacillus* [33, 34]. The pineal gland may be responsible for changes in gut microbiota [30]. Given this, we speculate that gut microbes may play a significant role in regulating the reproduction of hosts for adapting to the changing environment, but such studies are still lacking.

Brandt’s voles (*Lasiopodomys brandtii*) are a small herbivore species, which are widely distributed in the steppe grassland of the Mongolia plateau. They are typical seasonal breeders, with 2–4 litters per year and a breeding season from spring to autumn [35]. Brandt’s voles are key prey of many predators in the grassland [35, 36]. They dig and build complex burrow systems, which transfer fresh deep soil material to the surface and then accelerate nitrogen–carbon cycle of the grassland. Their underground tunnels provide nests or shelters for many invertebrate species, such as beetles and centipedes. Thus, they are keystone species in typical steppe for maintaining biodiversity and ecosystem function in a grassland ecosystem. Their annual population density oscillates greatly driven by both intrinsic (density-dependence) [37, 38] and external factors (rainfall and livestock grazing) [35, 39], and high-density population would cause damage to pastures [40]. It has been demonstrated that photoperiod affects the seasonal breeding of Brandt’s voles by regulating hypothalamic reproduction genes [2]. The peak of gonadal circadian rhythm in wild adult male voles appears around the summer solstice and is associated with changes in age structure [2].

Given the key role of photoperiod in regulating reproductive hormones [1–6] and some independent findings on the association of photoperiod with microbiota [30] and the association of microbiota with reproductive hormones [18–21, 41], our study aimed to investigate the association of the gut microbiota with photoperiodic regulation of seasonal breeding of Brandt’s voles. This may help us to better understand the mechanisms beneath population regulation and propose suitable management strategies for this species. We hypothesize that photoperiod may affect seasonal breeding by adjusting the composition of gut microbiota. We expect that (1) as compared to long-day (LD) photoperiod, short-day (SD) photoperiod reduces reproduction performance as characterized by a decrease in weight of reproductive organs (testis and epididymis), levels of serum hormones (FSH, LH, and T), hypothalamic genes (*Dio2*, *Rfrp-3*, and *Kiss-1*), and testicular genes (*Dio2*, *Kiss-1*, and *GPR54*), but increase in levels of serum hormone (MT) and testicular genes (*Dio3* and *Stra8*), and aggressive behavior, as demonstrated in various species [14, 42, 43]; (2) there is a significant difference in the composition of gut microbiota between SD and LD voles, which is associated with reproduction performance; and (3) implantation of specific gut microbiota into recipient voles can trigger similar photoperiod-induced changes in the performance of reproduction and behavior compared with those in SD and LD donor voles.

## Materials and methods

### Experimental animals

Adult male Brandt’s voles (2–3 months of age) were group-housed (2 individuals/cage) in polycarbonate cages (30 × 15 × 20 cm) at a room temperature of 23 ± 1°C under a long-day condition (light: dark = 14h: 10h) with free access to food (standard rabbit pellets, Beijing KeAo Bioscience Co.) and water in a breeding colony. All operations and procedures for animal raising and handling were conducted in line with guidelines of the Animal Care and Use Committee of the Institute of Zoology, Chinese Academy of Sciences. 

### Experiment 1

**Fig. 1:**
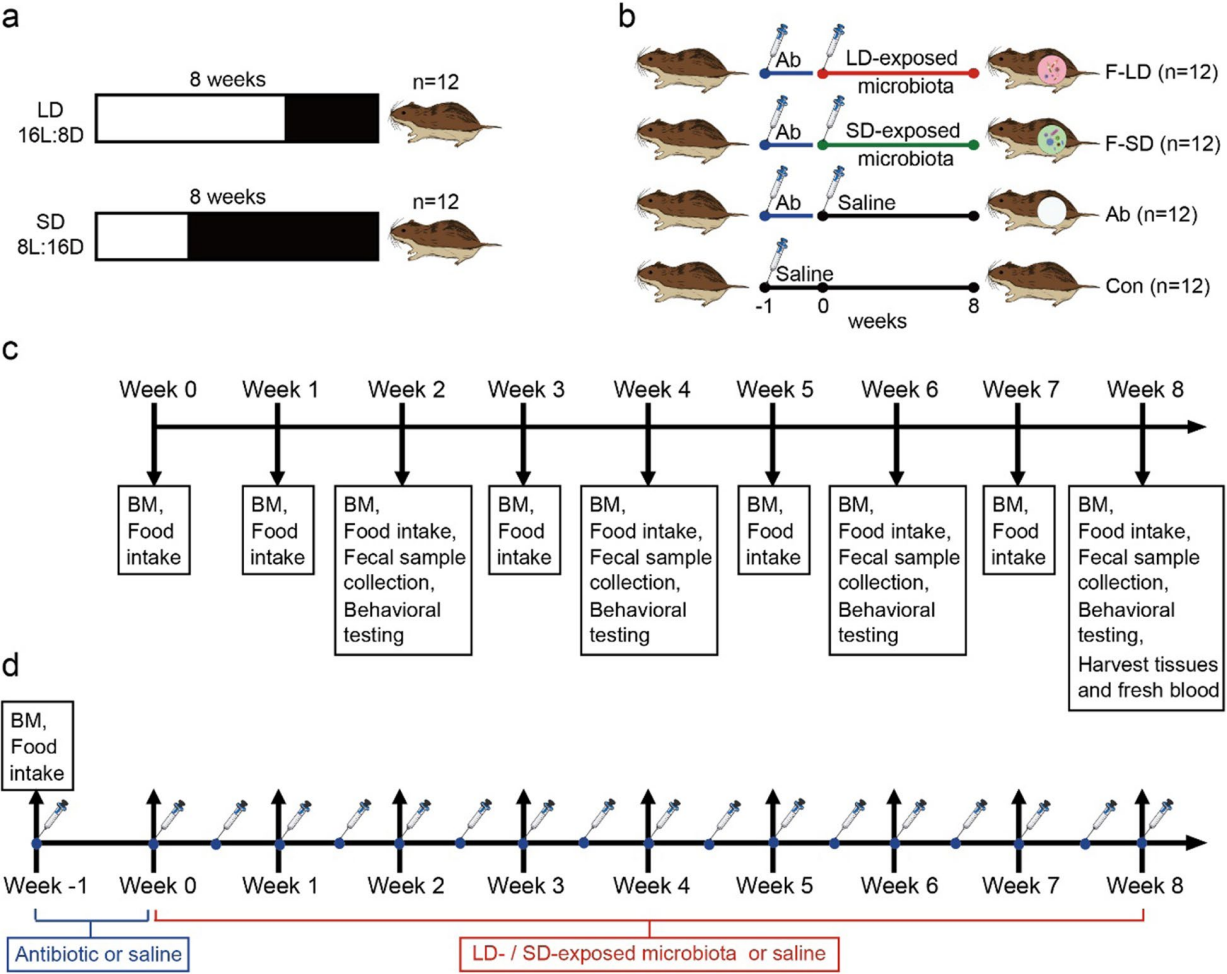
Overview of experimental design using Brandt’s voles. **a** Schematic diagram of photoperiod experiment. LD, long-day photoperiod (16L: 8D); SD, short-day photoperiod (8L: 16D). **b** Schematic diagram of FMT experiment. Con, recipients with saline; Ab, recipients with antibiotic; F-LD, recipients with LD-exposed microbiota; F-SD, recipients with SD-exposed microbiota. **c** Timeline of data collection for Experiment 1. **d** Timeline of data collection for Experiment 2. All measurements in Experiment 2 (corresponding to those as directed by up-arrows) were the same as those in Experiment 1. BM, body mass

Experiment 1 (i.e., the photoperiod experiment) was designed to test how physiological phenotypes, behavior, and gut microbiota of Brandt’s voles respond to LD and SD photoperiod treatments (Fig. 1a), aiming to test the first and second predictions. Twenty-four adult male voles were randomly divided into two groups (LD and SD groups; *n* = 12 per group). During the first 1 week (week 0), all voles were reared under a photoperiod of 14L: 10D prior to the start of the experiment. After 1 week of the adaptation period, voles in the LD and SD groups were transferred to and kept under a long-day condition (LD, light: dark = 16h: 8h) and short-day condition (SD, light: dark = 8h: 16h) for next 8 weeks, respectively. They were both kept in a room with a temperature of 23 ± 1°C, a light intensity of 200–300 lux, and a relative humidity of 55 ± 5%. The work flow of Experiment 1 was shown in Fig. 1c. During the photoperiod experiment, we measured body mass on the last day of every week and food intake on the last 2 days of every week. We measured aggressive behavior (from 08:00 to 10:00 a.m.) on the last day of every 2 weeks by using video cameras. On the last day of the experiment (week 8), we measured the locomotion behavior (representing the circadian rhythm) from 11:00 a.m. to the next 11:00 a.m. (24 h). We collected fecal samples (from 10:00 to 12:00 a.m.) on the last day of every 2 weeks. Fresh feces used for the fecal microbiota transplantation (FMT) experiment were collected on the last day of week 8. After the photoperiod experiment, voles were anesthetized and decapitated within 2 h to obtain fresh blood, hypothalamus, bilateral testes, and bilateral epididymis. Bilateral testis and epididymis were weighed. Centrifuged serum and tissues were placed at −80°C for further analysis (Fig. 1c).

### Experiment 2

Experiment 2 (i.e., the FMT experiment) was conducted to investigate the changes and associations of physiological phenotypes, behavior, and gut microbiota in recipient voles after microbiota transplantation from LD- and SD-photoperiod donor voles (Fig. 1b), aiming to test the third prediction. The work flow for Experiment 2 was shown in Fig. 1d. Donor voles were from the LD and SD groups (*n* = 12 for each group) at week 8. The feces (100 mg) from donors were dissolved in 2-mL sterile 0.9% saline and fully mixed. The supernatant was centrifuged (500 g, 1 min) to prepare a bacterial suspension. Forty-eight adult male voles were randomly divided into four groups (*n* = 12 for each group): a control group which received 200 μL sterile 0.9% saline by gavage all the time (Con), three groups in which voles were transplanted with 200 μL sterile saline (Ab), 200 μL LD-exposed microbiota (F-LD), and 200 μL SD-exposed microbiota (F-SD) for 8 weeks (from week 0 to week 8, twice a week, Fig. 1d) after successive 7-day administration of 200 μL antibiotics (50 μg/mL streptomycin, 100 μg/mL neomycin, and 100 U/mL penicillin; Sigma, Germany) via intragastric gavage from week −1 to week 0 (Fig. 1d), respectively. Voles were reared in a room with a temperature of 23 ± 1°C, a photoperiod of 14L: 10D, a light intensity of 200–300 lux, and a relative humidity of 55 ± 5%. During week 0 to week 8, the work flow and all measurements on body mass, food intake, behaviors, feces, tissues, and blood for Experiment 2 (Fig. 1d) were the same as those of Experiment 1 (Fig. 1c).

### Body mass and food intake measurement

During the acclimation period of both Experiment 1 and Experiment 2, all voles were weighed at 09:00 a.m. on the last day of every week by using an electronic balance (Sartorius Model BL 1500, precision 0.1g). We measured the food intake of each cage on the last 2 days of every week. We provided voles in each cage with 50 g of standard rabbit pellets at 10:00 a.m., and the remaining food was collected and weighed after 48 h (Fig. 1c, d). Food intake (g) of two voles per cage was calculated by subtracting the weight of unconsumed food from the initial food weight. After food intake measurement, all left food fragments were removed and replaced with fresh food.

### Behavior measurement

We used video cameras (Canon, LEGRIA HF-806) to record the aggressive behavior of voles in both Experiment 1 and Experiment 2 from 10:00 to 12:00 a.m. at the last day of weeks 2, 4, 6, and 8 (Fig. 1c, d). By following Takahashi and Miczek (2014) [44], we defined aggressive behavior as a reciprocal attack or fighting of the two voles in the cage. The intensity of the attack was represented by the number of attacks (total number of attacks within 2 h) and attack duration (cumulative time of attack within 2 h) of voles. On the last day of week 8 (from 11:00 a.m. to the next 11:00 a.m.; 24 h), we recorded the locomotion activity representing the circadian rhythms of voles for 24 h by using video cameras. We defined locomotion activity as voluntary movement or motion of voles from one place to another in the cage. We took every 30 min as a time frame to calculate the cumulative time of locomotion activity of each vole and obtained 48 values within 24 h to describe the circadian rhythm of voles. The proportion of locomotion activity time for each 30 min time window was calculated by dividing the cumulative time of locomotion activity within 30 min time window by the total cumulative locomotion time within the 24 h.

### Fecal sampling and tissue collection

We collected feces of voles in both Experiment 1 and Experiment 2 by placing each of 2 voles in a sterile container (26 × 12 × 12 cm) with a paper under the containers between 08:00 a.m. and 10:00 a.m. on the last day of weeks 2, 4, 6, and 8. Fresh feces were collected from each vole within 10 min with forceps and then stored at −80°C for subsequent analysis [45]. After sample collection, the voles were returned to their own cages.

By the end of both Experiment 1 and Experiment 2 at week 8, voles were anesthetized by intraperitoneal injection of 0.6% sodium pentobarbital (60 mg/kg) from 08:00 to 10:00 a.m. and sacrificed by decapitation. The hypothalamus, bilateral testes, and bilateral epididymis of the voles were immediately removed. Bilateral testis and epididymis were weighed. Trunk blood was collected immediately after decapitation (less than 2 min) and then left at room temperature for 2 h and centrifuged at 3000 rpm for 20 min under 4°C. The collected serum samples were stored at −80°C until further processing.

### Serum MT, FSH, LH, GnRH, and T levels assays

Enzyme-linked immunosorbent assay (ELISA) kit (specific for mice; Jianglai Bio., China) was used to determine the serum MT, FSH, LH, GnRH, and T levels in donor and recipient voles. The detection range of the MT, FSH, LH, GnRH, and T kits were 2.5–80 pg/mL, 2.5–80 mIU/mL, 0.25–8 mIU/mL, 2.5–80 mIU/mL, and 0.25–8 ng/mL, respectively. Their minimum detectable concentrations were 0.1 pg/mL, 0.1 mIU/mL, 0.1 mIU/mL, 0.1 mIU/mL, and 0.1 ng/mL. The intra- and inter-assay coefficients of variation (CV) of all kits for detecting serum hormones were less than 10% and 15%, respectively.

### RNA isolation and quantitative real-time PCR (qRT-PCR)

Total RNA was extracted from the hypothalamus and right testis of donor and recipient voles (*n* = 12 per group) using RNAiso Plus kit (TaKaRa, Otsu, Japan). The quantity of total RNA was assessed by 1% agarose gel electrophoresis (*U* =120 V; 10 min). The concentration of RNA was measured using the Nano-100 (Allsheng, Hangzhou, China). The 260/280 ratio of 1.8 to 2.0 indicated that the quality of the total RNA isolated is high. Total RNA (200 ng) was reversely transcribed into 20 μL cDNA using the PrimeScript RT kit (TaKaRa, Otsu, Japan).

Partial- or full-length cDNA sequences of existing rodents *Dio2*, *Dio3*, *Kiss-1*, *GPR54*, *GnRH*, *Rrfp*-3, *Stra8*, and *β-actin* were acquired from GenBank, and we designed specific primers from the conserved regions of known sequences mentioned above for qRT-PCR via Primer 5.0 according to Chen et al. [46], Liu et al. [47], and Wang et al. [2] (Table S1). The thermal cycle conditions of qRT-PCR were as follows pre-denaturation at 94°C for 5 min, followed by 42 cycle denaturation at 94°C for 10 s, annealing at 62°C for 15 s, extension at 72°C for 20 s, and then extension at 72°C for 10 min. Each gene sample of the two experiments was run in triplicate and obtained threshold values. The amplification efficiency of the target gene and the reference gene (*β-actin*) were both close to 100% and the relative deviation did not exceed 5%, and the relative expression levels of the target gene were analyzed by the 2^−ΔΔCT^ method.

### DNA extractions and 16S rRNA gene sequencing analysis

Genomic DNA was extracted from feces of donor and recipient voles by the 2 × cetyltrimethylammonium bromide (CTAB) method, and then, the purity and concentration of DNA were measured by 1% agarose gel electrophoresis. An appropriate amount of sample was taken into the centrifuge tube and was diluted to 1 ng/μL with sterile water. According to Zhang et al. [48], we used forward primer 341F (5′-CCTAYGGGRBGCASCAG-3′) and reverse primer 806R (5′-GGACTACNNGGGTATCTAAT-3′) to amplify the V3–V4 regions of 16S rRNA gene of colon fecal microbiota. The libraries were established and added with index codes using the NEB Next® Ultra™ DNA Library Prep Kit (New England Biolabs, USA) for Illumina. The quality of the library was evaluated using a Qubit@ 2.0 Fluorometer (Thermo Scientific). Finally, the Illumina HiSeq 2500 platform was used for sequencing and generating 250 bp paired-end reads.

Raw sequences were processed using Quantitative Insights Into Microbial Ecology (QIIME, version 1.9.1) software. Paired-end sequences were merged using Flash-1.2.8 (V1.2.8, http://ccb.jhu.edu/software/FLASH/). The merged high-quality sequences for subsequent analysis included (1) chloroplast sequences were removed using Metaxa 2 software, (2) VSEARCH with SILVA database was used to remove chimeric sequences, (3) sequence data were processed through the Unoise3 pipeline in R to infer amplicon sequence variants (ASVs) [49], and (4) finally, we annotated taxonomic information using Ribosomal Database Project (RDP) Database within QIIME. We used the single_rarefaction.py script in the QIIME pipeline to normalize the read to 33,074 for each sample of the photoperiod experiment and to 41,556 for each sample of the FMT experiment to eliminate the effect of sequencing depth. We used R software (version 4.1.1) to display and used QIIME to calculate alpha diversity (the Richness index, ACE index, and Shannon index) of the intestinal microbial communities and to establish rarefaction curves with Richness index. Based on the Bray-Curtis dissimilarity distances, constrained principal coordinates analysis (CPCoA) was used to estimate the difference in beta diversity at the ASVs level. We used linear discriminant analysis (LDA) effect size (LEfSe) [50] to evaluate the differences in gut microbiota and to search for biomarkers with statistical differences between groups.

### Statistical analysis

Levene tests and Shapiro-Wilk tests were used to assess variance and normality of food intake and aggressive behavior data for both Experiment 1 and Experiment 2, respectively. And repeated-measures analysis of variance (ANOVA) model was used to analyze the difference in food intake and aggressive behavior using SPSS 20.0. Body mass, genital organs, reproductive hormones (serum MT, FSH, LH, GnRH, and T levels), gene expression in hypothalamus (*Dio2*, *Kiss-1*, *GPR54*, *GnRH*, and *Rfrp-3*) and testis (*Dio2*, *Dio3*, *Dio2/Dio3*, *Kiss-1*, *GPR54*, *GnRH*, and *Stra8*), and alpha diversity indices for both Experiment 1 and Experiment 2 were all assessed by linear mixed model (LMM) with treatment as the fixed factor, and housing cage as the random factor in R software (version 4.1.1). For non-normally distributed variables, Box-Cox transformation was used to fit model assumption. Spearman correlation analysis was used to analyze the correlation between the relative abundance of gut microbiota and levels of hormones or genes expression for both Experiment 1 and Experiment 2 in R software (version 4.1.1). Permutational multivariate analysis of variance (PERMANOVA) was used to determine whether treatment and different weeks affected significant differences in beta diversity based on Bray-Curtis distance (adonis function in R, permutation = 9999) for both Experiment 1 and Experiment 2 [51]. Differences in circadian behavioral rhythm patterns between treatment groups were evaluated using PERMANOVA (adonis with Bonferroni’s post hoc tests) for both Experiment 1 and Experiment 2. Figures were performed in GRAPH PRISM 7.0 and RStudio. Data are presented as means ± SEM and significance levels were set at *P* < 0.05.

## Results

### Different responses between the LD and SD groups in the photoperiod experiment

#### Effect of photoperiod on behaviors, food intake, and body mass

**Fig. 2:**
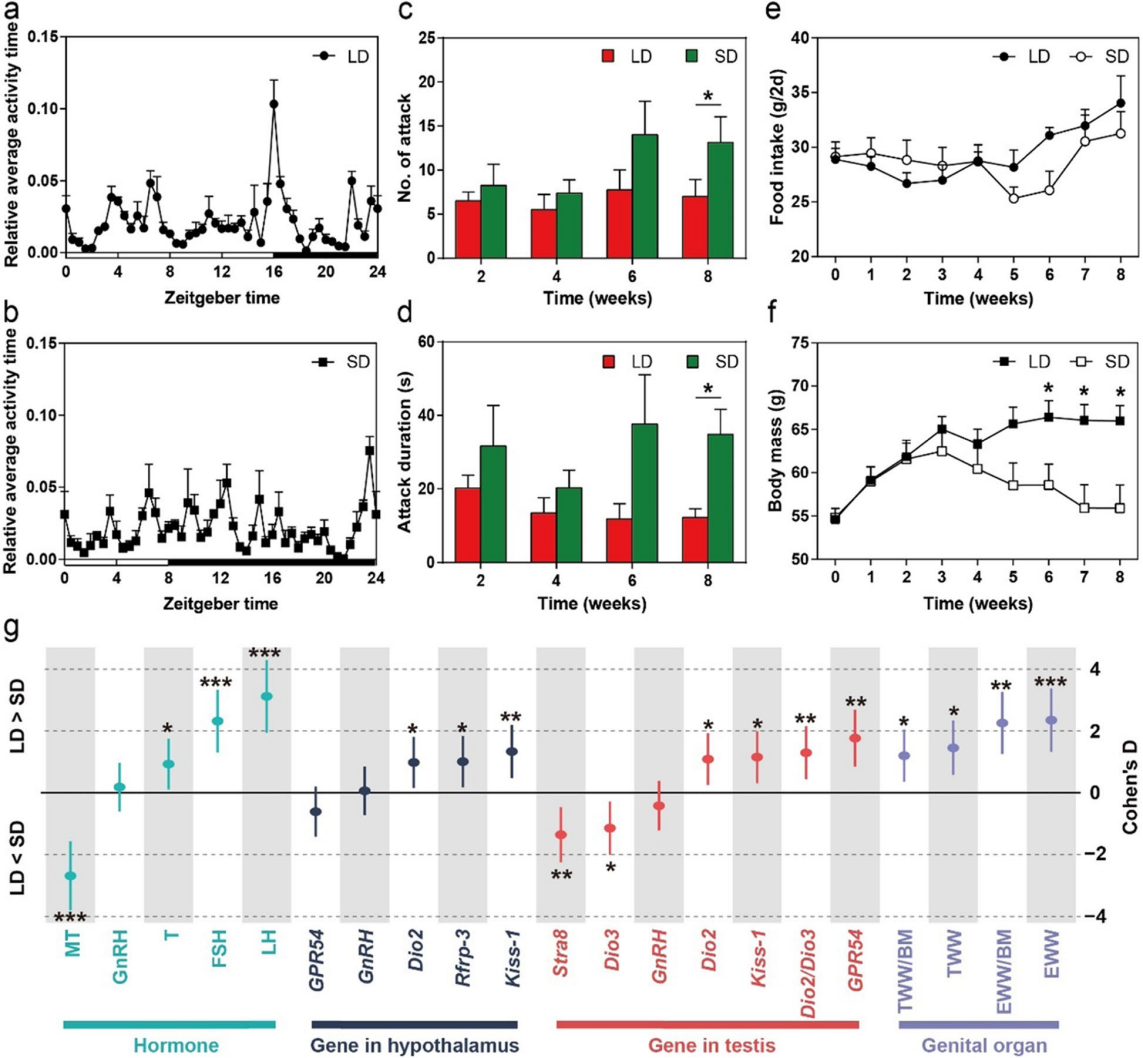
Photoperiod-induced alterations in behaviors, body mass, food intake, and reproductive phenotypes of Brandt’s voles. **a**, **b** Relative average activity time of voles in 24 h by the end of photoperiod experiment. **c**, **d** Number of attacks and attack duration at week 2, week 4, week 6, and week 8. **e**, **f** Changes in food intake and body mass during the period of photoperiod acclimation. **g** Effect size (i.e., Cohen’s D) of photoperiod manipulation on reproductive hormones or genes expression. Cohen’s D = (Mean_LD_–Mean_SD_)/pooled standard deviations for the two groups. TWW, testicular wet weight; TWW/BM, the ratio of testicular wet weight to body mass; EWW, epididymis wet weight; EWW/BM, the ratio of epididymis wet weight to body mass. *P < 0.05, **P < 0.01, and ***P < 0.001

Photoperiod significantly affected behaviors, food intake, and body mass of Brandt’s voles. In the LD group, the highest peak of circadian rhythm of voles appeared before dark period (Fig. 2a), while in the SD group, the peak appeared before dawn (Fig. 2b). PERMANOVA revealed significant differences in circadian behavioral patterns between the LD and SD groups (*F*_1,22_ = 1.15, *P* = 0.003). Repeated-measures ANOVA analysis showed that number of attacks (*t* = −2.19, *P* = 0.046; Fig. 2c) and attack duration (*t* = −3.14, *P* = 0.007; Fig. 2d) in the SD group were significantly higher than those in the LD group at week 8. In addition, there was no significant difference in food intake between two groups during photoperiod acclimation (*F*_1,10_ = 0.25, *P* = 0.6; Fig. 2e). Compared to the SD group, linear mixed model (LMM) analysis showed that body mass of the LD group increased over time and significantly increased in the last three weeks (week 6: *t* = 2.24, *P* = 0.048; week 7: *t* = 2.72, *P* = 0.02; week 8: *t* = 2.81, *P* = 0.02; Fig. 2f).

#### Effect of photoperiod on reproductive hormones and genes

LMM analysis revealed that photoperiod had a significant effect on the serum concentrations of MT (*F*_1,10_ = 43.27, *P* < 0.001), FSH (*F*_1,10_ = 27.42, *P* < 0.001), LH (*F*_1,10_ = 58.43, *P* < 0.001), and T (*F*_1,10_ = 5.13, *P =* 0.047), but had no effect on serum GnRH concentration (*F*_1,10_ = 0.20, *P =* 0.7). The concentration of MT in the SD group was significantly higher than that in the LD group, while the concentrations of FSH, LH, and T in the SD group were significantly lower than those in the LD group. Several mRNA levels of genes, such as *Dio2* (*F*_1,10_ = 5.78, *P =* 0.04), *Rfrp-3* (*F*_1,10_ = 6.07, *P =* 0.03), and *Kiss-1* (*F*_1,10_ = 10.71, *P =* 0.008) in the hypothalamus, were significantly upregulated by LD photoperiod, while the other mRNA levels of genes such as *GPR54* (*F*_1,10_ = 1.98, *P =* 0.2) and *GnRH* (*F*_1,10_ = 0.02, *P =* 0.9) mRNA showed no difference in expression between two groups. In addition, in the testis, the expression levels of *Dio2* (*F*_1,10_ = 7.11, *P =* 0.02), *Dio2/Dio3* (*F*_1,10_ = 10. 06, *P* = 0.01), *Kiss-1* (*F*_1,10_ = 7.99, *P =* 0.02), and *GPR54* (*F*_1,10_ = 18.74, *P* = 0.001) in the LD group were significantly higher than those in the SD group, whereas the expression levels of *Dio3* (*F*_1,10_ = 7.82, *P =* 0.02) and *Stra8* (*F*_1,10_ = 11.03, *P =* 0.01) of the LD group were significantly lower than the SD group. *GnRH* expression (*F*_1,10_ = 0.85, *P =* 0.4) did not show significant differences between two groups in the testis. Moreover, testicular wet weight (*F*_1,10_ = 9.55, *P =* 0.01), the ratio of testicular wet weight to body mass (*F*_1,10_ = 6.63, *P =* 0.03), epididymis wet weight (*F*_1,10_ = 23.33, *P* < 0.001), and the radio of epididymis wet weight to body mass (*F*_1,10_ = 18.14, *P* = 0.002) in the LD group were significantly higher than those in the SD group (Fig. 2g).

#### Effect of photoperiod on gut microbiota

**Fig. 3:**
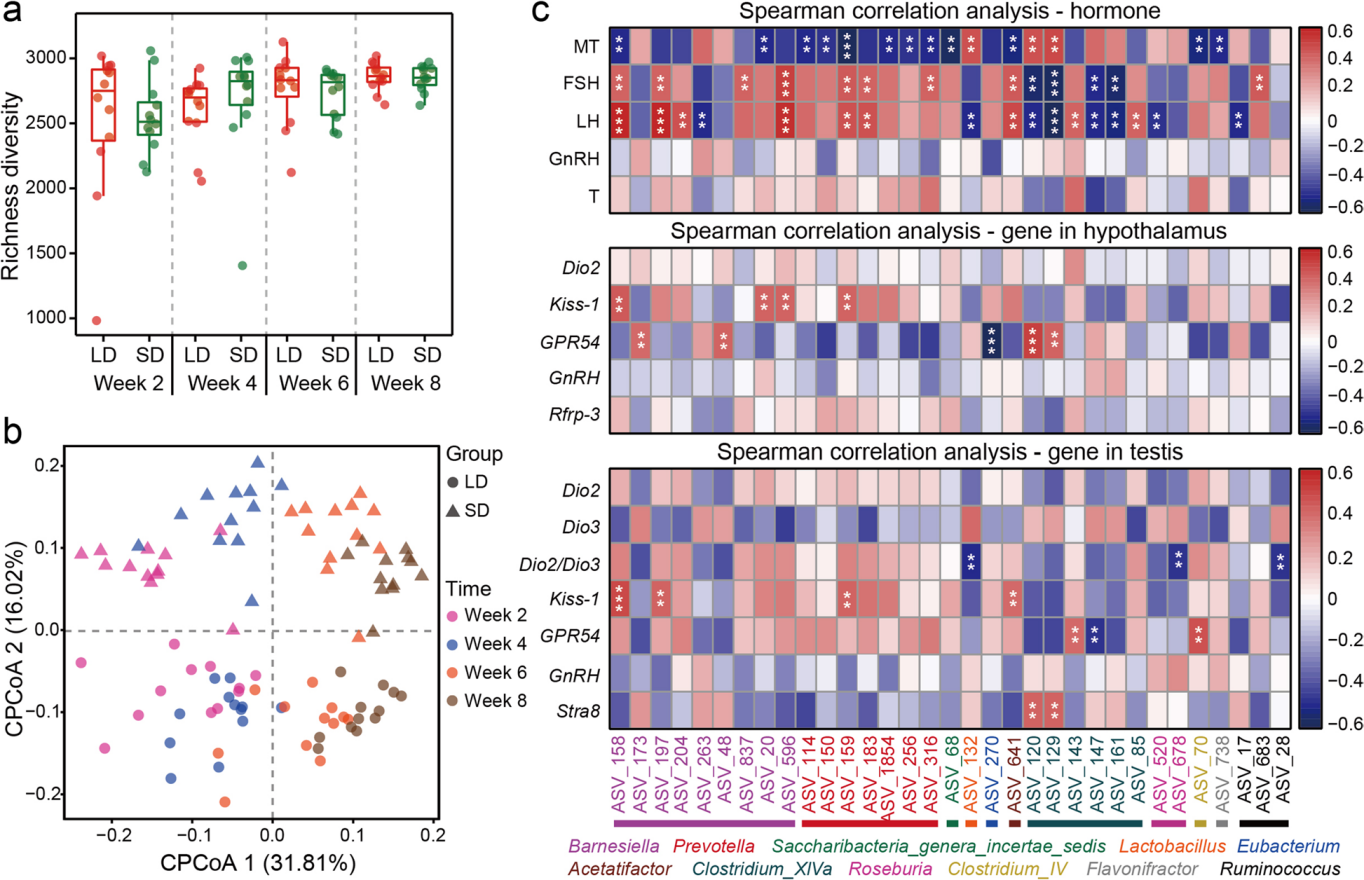
Fecal microbiota in the LD and SD groups and their relationship with reproductive phenotypes. **a** Richness diversity (means ± SEM) of bacterial communities across groups. **b** Constrained principal coordinate analysis (CPCoA) based on the Bray-Curtis dissimilarity matrix at the amplicon sequence variants (ASVs) level in different groups. **c** Heatmap showing Spearman correlation between ASVs and serum hormone levels or gene expression in the hypothalamus and testis. Only the ASVs that had a highly significant difference between photoperiod treatments (*P* < 0.01) were labeled with asterisks: **P < 0.01 and ***P < 0.001

Richness diversity rarefaction curve for all samples reached to stable values (Figure S1a), indicating that the sequencing depth was sufficient to represent the bacterial diversity of the samples. Alpha diversity indices of voles were significantly affected by different weeks (LMM, Richness diversity: *F*_3,27.15_ = 3.78, *P =* 0.02; Fig. 3a; Shannon diversity: *F*_3,27.21_ = 4.58, *P* = 0.01; Figure S1b; ACE diversity: *F*_3,26.37_ = 6.21, *P* = 0.002; Figure S1c), but not affected by photoperiod treatments (Richness diversity: *F*_1,10_ = 0.015, *P =* 0.9; Fig. 3a; Shannon diversity: *F*_1,10_ = 0.8, *P =* 0.4; Figure S1b; ACE diversity: *F*_1,10_ = 0.003, *P =* 0.95; Figure S1c). Constrained principal coordinate analysis (CPCoA) based on the Bray-Curtis dissimilarity matrix showed that samples were distinctly separated, with samples in the SD group occupying the upper half axis, and samples in the LD group all being located in the lower half axis (Fig. 3b). The PERMANOVA showed significant differences in gut microbiota community between the LD and SD groups at week 4 (*F*_1,22_ = 1.43, *P* = 0.04), at week 6 (*F*_1,22_ = 2.00, *P* = 0.001), and at week 8 (*F*_1,22_ = 2.07, *P* = 0.001), whereas no significant difference was found at week 2 (*F*_1,22_ = 1.38, *P* = 0.07).

LEfSe analysis indicated that there were significant differences in the composition of microbial communities between the LD and SD groups at weeks 2, 4, 6, and 8 (Figure S1d). At week 8, at the phylum levels, bacteria of phylum *Firmicutes* were significantly enriched in the SD group. At lower phylogenetic levels, bacteria of six discriminant clades (class *Bacilli*, order *Lactobacillales*, families *Lactobacillaceae* and *Lachnospiraceae*, and genera *Ruminococcus* and *Lactobacillus*) were enriched in the SD group. In contrast, bacteria of the other four discriminant clades (class *Bacteroidia*, order *Bacterodia*, family *Rikenellaceae*, and genus *Alistipes*) were enriched in the LD group.

Heatmap showed those altered ASVs by photoperiod were also associated with serum hormones levels and gene expression in hypothalamus and testis (Fig. 3c). For example, the relative abundances of *Barnesiella*, *Prevotella*, *Acetatifactor*, and *Clostridium_XIVa* were correlated with serum MT, FSH, and LH levels. The relative abundances of *Saccharibacteria_geneta_incertae_sedis*, *Clostridium*_*IV*, and *Flavonifractor* were correlated with MT levels in serum; *Lactobacillus* was correlated with MT and LH levels in serum; *Roseburia* was correlated with LH levels in serum; and *Ruminococcus* was correlated with FSH and LH levels in serum (all *P* < 0.01; Table S2). In hypothalamus, there were significant correlations between mRNA expression of several genes and gut microbes: *Kiss-1* mRNA with *Barnesiella* and *Prevotella*; *GPR54* mRNA with *Barnesiella*, *Eubacterium*, and *Clostridium_XIVa* (all *P* < 0.01; Table S3). In the testis, there were significant correlations between mRNA expression of several genes and gut microbes: *Dio2/Dio3* mRNA with *Lactobacillus*, *Roseburia*, and *Ruminococcus*; *Kiss-1* mRNA with *Barnesiella*, *Prevotella*, and *Acetatifactor*; *GPR54* mRNA with *Clostridium_XIVa* and *Clostridium*_*IV*; *Stra8* mRNA with *Clostridium_XIVa* (all *P* < 0.01; Table S4, S5). These results indicate that photoperiod-induced changes in gut microbiota composition were closely related to reproductive hormones and genes during photoperiod adaptation.

### Different responses between the F-LD and F-SD groups in the FMT experiment

#### Effects of FMT on behavior, food intake, and body mass

**Fig. 4:**
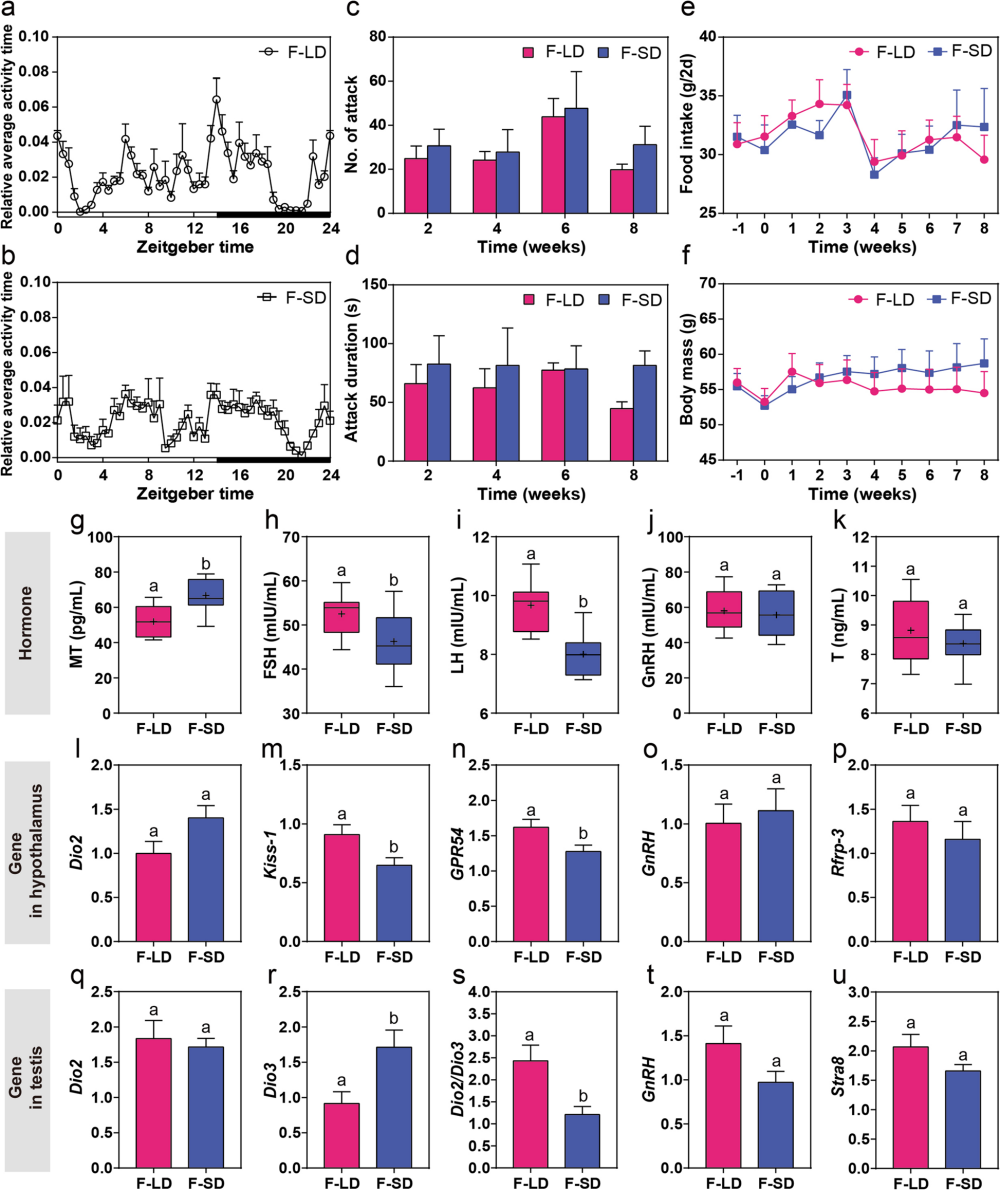
Effects of FMT on behaviors and reproductive phenotypes of Brandt’s voles. **a**, **b** Relative average activity time of voles in 24 h at the end of FMT treatment. **c**, **d** Number of attacks and attack duration at week 2, week 4, week 6, and week 8. **e**, **f** Changes in food intake and body mass during the period of FMT treatment. **g–k** MT, FSH, LH, GnRH, and T levels in serum. **l**–**p** The expression of *Dio2*, *Kiss-1*, *GPR54*, *GnRH*, and *Rfrp-3* in the hypothalamus. **q**–**u** The expression of *Dio2*, *Dio3*, *Dio2/Dio3*, *GnRH*, and *Stra8* in the testis

FMT experiment results indicated that the circadian rhythm peaks of both the F-LD and F-SD groups appeared before the dark period, and the circadian rhythm peaks of the F-SD group were lower than those of the F-LD group (Fig. 4a, b). PERMANOVA revealed no difference in circadian behavioral patterns between the F-LD and F-SD groups (*F*_1,22_ = 0.88, *P* = 0.5). Number of attacks (*F*_1,10_ = 0.66, *P* = 0.4; Fig. 4c) and attack duration (*F*_1,10_ = 1.56, *P* = 0.2; Fig. 4d) showed no difference between two groups, although F-SD voles showed tendency of high aggression. In addition, no difference at food intake (*F*_1,10_ = 0.06, *P* = 0.8; Fig. 4e) or body mass (*F*_1,10_ = 0.15, *P* = 0.7; Fig. 4f) was observed between F-LD and F-SD voles.

#### Effects of FMT on reproductive hormones and genes

For FMT experiment, LMM analysis showed that serum MT levels in the F-SD group were significantly higher than those in the F-LD group (*F*_1,10_ = 11.74, *P* = 0.006; Fig. 4g), while serum FSH (*F*_1,10_ = 5.06, *P* = 0.048; Fig. 4h) and LH (*F*_1,10_ = 27.79, *P* < 0.001; Fig. 4i) levels in the F-SD group were significantly lower than those in the F-LD group. Serum GnRH (*F*_1,10_ = 0.23, *P* = 0.6; Fig. 4j) and T (*F*_1,10_ = 1.35, *P* = 0.3; Fig. 4k) concentrations showed no significant change between two groups. In the hypothalamus, the relative expression levels of *Kiss-1* (*F*_1,10_ = 5.49, *P* = 0.04; Fig. 4m) and *GPR54* (*F*_1,10_ = 5.67, *P* = 0.04; Fig. 4n) in the F-LD group increased significantly compared to the F-SD group, whereas *Dio2* (*F*_1,10_ = 2.30, *P* = 0.1; Fig. 4l), *GnRH* (*F*_1,10_ = 0.16, *P* = 0.7; Fig. 4o), and *Rfrp-3* (*F*_1,10_ = 0.56, *P* = 0.5; Fig. 4p) expression showed no significant differences between two groups. In the testis, *Dio3* mRNA levels (*F*_1,10_ = 6.08, *P* = 0.03; Fig. 4r) increased, whereas *Dio2/Dio3* ratio (*F*_1,10_ = 11.74, *P* = 0.04; Fig. 4s) decreased in F-SD voles compared to F-LD voles. There was no significant difference in the expression levels of *Dio2* (*F*_1,10_ = 0.15, *P* = 0.7; Fig. 4q), *GnRH* (*F*_1,10_ = 3.01, *P* = 0.1; Fig. 4t), *Stra8* (*F*_1,10_ = 2.19, *P* = 0.2; Fig. 4u), *Kiss-1* (*F*_1,10_ = 0.073, *P* = 0.8; Figure S2a), and *GPR54* (*F*_1,10_ = 0.22, *P* = 0.7; Figure S2b) between two groups in the testis. Testicular wet weight (*F*_1,10_ = 1.45, *P* = 0.3; Figure S2c), the ratio of testicular wet weight to body mass (*F*_1,10_ = 0.51, *P* = 0.5; Figure S2d), epididymis wet weight (*F*_1,10_ = 0.11, *P* = 0.7; Figure S2e), and the radio of epididymis wet weight to body mass (*F*_1,10_ = 0.04, *P* = 0.8; Figure S2f) did not show significant differences between two groups.

#### Effect of FMT on gut microbiota

**Fig. 5:**
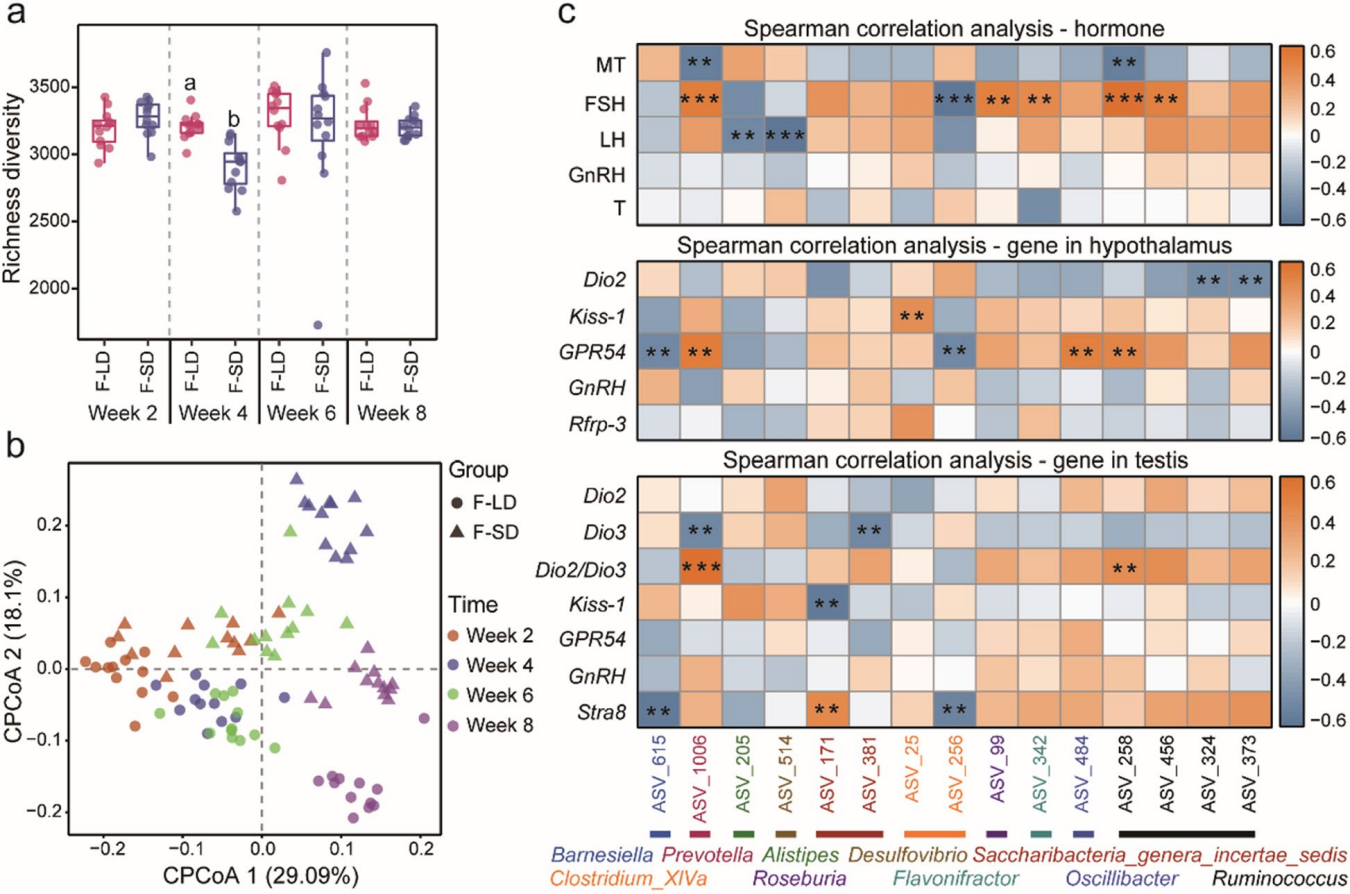
Fecal microbiota in the F-LD and F-SD groups and their relationship with reproductive phenotypes. **a** Richness diversity (means ± SEM) of bacterial communities across two groups. **b** Constrained principal coordinate analysis (CPCoA) plot based on Bray-Curtis distance metrics shows the microbial community structure of samples from the F-LD and F-SD groups. **c** Heatmap showing Spearman correlation between ASVs and serum hormone levels or gene expression in the hypothalamus and testis. Only the ASVs that had a highly significant difference between the two groups (*P* < 0.01) were labeled with asterisks: **P < 0.01 and ***P < 0.001

Richness diversity rarefaction curve for all samples reached to stable values (Figure S2g), indicating that most of the microbial diversity had been captured in FMT experiment. LMM analysis showed that both different weeks and FMT treatment affected Richness diversity (time: *F*_3,23.94_ = 3.47, *P* = 0.03; treatment: *F*_1,72.87_ = 4.39, *P* = 0.04; Fig. 5a) and ACE diversity (time: *F*_3,23.94_ = 6.96, *P* = 0.002; treatment: *F*_1,72.87_ = 5.39, *P* = 0.02; Figure S2i), whereas there was no difference in Shannon diversity (time: *F*_3,25.31_ = 0.38, *P* = 0.8; treatment: *F*_1,72.87_ = 0.78, *P* = 0.4; Figure S2h). Furthermore, the three indices in the F-SD group were significantly lower than those in the F-LD group at week 4 (Richness diversity: *F*_1,22_ = 25.75, *P* < 0.001; ACE diversity: *F*_1,22_ = 48.17, *P* < 0.001; Shannon diversity: *F*_1,22_ = 8.92, *P* = 0.007). Beta diversity based on Bray-Curtis distance by CPCoA, FMT treatment was distinctly separated between the F-LD and F-SD groups, with samples of the F-SD group being located in the upper half axis and samples of the F-LD group all being located in the lower half axis (Fig. 5b). PERMANOVA revealed differences in microbial communities between two groups at week 2 (*F*_1,22_ = 2.04, *P* < 0.001), week 4 (*F*_1,22_ = 3.18, *P* < 0.001), week 6 (*F*_1,22_ = 1.7, *P* = 0.007), and week 8 (*F*_1,22_ = 1.74, *P* < 0.001).

LEfSe method analysis identified different microbial communities in the F-LD and F-SD groups (Figure S2j). In week 8, compared with the F-LD group, at the phylum level, *Verrucomicrobia* was significantly more enriched in the F-SD group; at the class level, *Verrucomicrobiae* was higher in the F-SD group; at the order class, *Verrucomicrobiales* increased in the F-SD group; at the family level, the relative abundance of *Porphyromonadaceae* and *Verrucomicrobiaceae* were significantly higher in the F-SD group; at the genus level, the proportions of A*kkermansia* increased in the F-SD group.

The heatmap showed the correlation between hormone and gene expression and the relative abundance of differential ASVs in the F-LD and the F-SD groups (Fig. 5c). The genera of *Prevotella* and *Ruminococcus* were correlated with serum MT levels; *Prevotella*, *Clostridium_XIVa*, *Roseburia*, *Flavonifractor*, and *Ruminococcus* were correlated with FSH levels in serum; *Alistipes* and *Desulfovibrio* were correlated with LH concentration (all *P <* 0.01; Table S6). In the hypothalamus, there were significant correlations between mRNA levels of several genes and gut microbes: the expression of *Dio2* with *Ruminococcus*; the expression of *Kiss-1* with *Clostridium_XIVa*; and the expression of *GPR54* with *Barnesiella*, *Prevotella*, *Clostridium_XIVa*, *Oscillibacter*, and *Ruminococcus* (all *P <* 0.01; Table S7). In the testis, *Barnesiella* was correlated with *Stra8* mRNA; *Prevotella* was correlated with *Dio3* and *Dio2*/*Dio3* ratio; *Saccharibacteria*_*genera*_*incertae*_*sedis* was correlated with *Dio2*/*Dio3* ratio, *Kiss-1*, and *Stra8* mRNA; *Clostridium_XIVa* was correlated with *Stra8* mRNA; and *Ruminococcus* was correlated with *Dio2*/*Dio3* ratio (all *P <* 0.01; Table S8, S9).

### Different responses between the control (Con, Ab) and FMT (F-SD, F-LD) groups in the FMT experiment

#### Differences in body mass, food intake, and behaviors

There was no significant difference of body mass (*F*_3,20.01_ = 0.16, *P =* 0.9; Figure S3a) and food intake (*F*_3,20_ = 0.89, *P =* 0.5; Figure S3b) between four groups except that food intake in the F-LD group was significantly higher than that in the Con group at week 6 (*P =* 0.045).

There was no significant difference in number of attacks (*F*_3,20_ = 1.92, *P =* 0.2; Figure S3c) and attack duration (*F*_3,20_ = 1.61, *P =* 0.2; Figure S3d) between the control and FMT groups. The PERMANOVA revealed significant differences in circadian rhythms between the Ab and other three groups (Ab *vs* Con: *F*_1,22_ = 2.21, *P* = 0.01; Ab *vs* F-LD: *F*_1,22_ = 2.18, *P* = 0.01; Ab *vs* F-SD: *F*_1,22_ = 2.59, *P* = 0.004), while there was no such difference between the Con and F-LD groups (*F*_1,22_ = 1.87, *P* = 0.06) or between the Con and the F-SD groups (*F*_1,22_ = 1.32, *P* = 0.2). As compared to the Ab group, the circadian rhythm peak in the F-LD group appeared before the dark period, and the circadian rhythm peaks were lowered in the Con and F-SD groups (Figure S3e, f; Fig. 4a, b).

#### Differences in reproductive hormones and genes

LMM analysis showed that compared with the F-LD group, serum MT, LH, and GnRH levels, hypothalamic *GPR54*, testicular *Dio2*, *Dio2/Dio3*, *GnRH*, and *Stra8* mRNA were decreased but serum FSH levels was increased significantly in the Con group (Figure S4, Table S11, S12). There was no difference in other physiological indicators (hypothalamic *Dio2*, *Kiss-1*, *GnRH*, and *Rfrp-3* mRNA; testicular *D*io*3*, *Kiss-1*, and *GPR54* mRNA; testicular wet weight, the ratio of testicular wet weight to body mass, epididymis wet weight, and the ratio of epididymis weight to body mass). Compared with the F-LD group, the Ab group had a significant decrease in serum MT, LH, and T levels, but increase in serum FSH levels; there was no difference in other physiological indicators (Figure S4, Table S11, S12). Compared with the F-SD group, serum MT, LH, GnRH, and T levels, and testicular *Dio2* and *Dio3* mRNA were significantly decreased, while serum FSH levels and hypothalamic *Kiss-1* mRNA were significantly increased in the Con group; there was no difference in other physiological indicators (Figure S4, Table S11, S12). The levels of serum MT, LH, and T were significantly lower, while the levels of serum FSH, hypothalamic *Kiss-1* and *GPR54* mRNA, and testicular *Dio2/Dio3* mRNA were significantly higher in the Ab group than those of the F-SD group, and there was no difference in other physiological indicators (Figure S4, Table S11, S12).

#### Differences in gut microbiota

LMM analysis showed that both time and treatment affected alpha diversity between control groups and FMT groups (ACE diversity and Richness diversity all *P* < 0.05; Figure S5c, d, Table S13). There was a significant difference in beta diversity among the four groups, and the FMT groups were completely separated from the Con and Ab groups, with the samples of FMT groups all located in the upper half axis and the samples of the Con and Ab groups all located in the lower half axis (PERMANOVA, Figure S5e, Table S14). LEfSe method analysis revealed the significant differences in microbial communities among the four groups (Figure S5f).

### Comparisons of different responses between donor and recipient voles

#### Reproductive hormones and genes

**Fig. 6:**
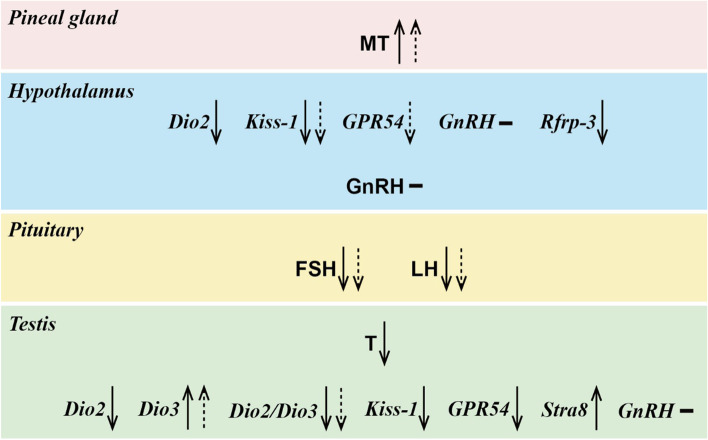
Summary on the changes of physiological indices in donor voles (solid arrow) and recipient voles (dashed arrow). Upward/down arrow shows an increase/decrease in hormone levels or gene expression under SD or F-SD conditions as compared to LD or F-LD treatment. “-” indicates no change. MT, melatonin; GnRH, gonadotropin-releasing hormone; FSH, follicle-stimulating hormone; LH, luteinizing hormone; T, testosterone; *Dio2*, iodothyronine deiodinase 2; *Dio3*, iodothyronine deiodinase 3; *Dio2/Dio3*, the ratio of *Dio2* to *Dio3* expression; *Kiss*-1, Kisspeptin-1; *GPR54*, G-protein-coupled receptor 54; *GnRH*, gene which encode gonadotropin-releasing hormone; *Rfrp*-3, RFamide-related peptide 3; *Stra8*, stimulated by retinoic acid 8. LD, long-day photoperiod (16L: 8D); SD, short-day photoperiod (8L: 16D); F-LD, recipients with transplantation of gut microbiota from LD donor voles; F-SD, recipients with transplantation of gut microbiota from SD donor voles

Compared to LD or F-LD voles, F-SD recipient voles showed similar response in three hormones (higher MT, lower FSH and LH) and three genes (lower *Kiss-1* in the hypothalamus, higher *Dio3* and *Dio2/Dio3* ratio in the testis) to those in SD donor voles (Fig. 6). In addition, F-SD recipient voles showed a unique effect on *GRP54* in the hypothalamus. The negative effect of SD voles on serum T levels, the expression of *Dio2* and *Rfrp-3* in the hypothalamus, *Dio2*, *Kiss-1*, and *GRP54* in the testis, and positive effect *Stra8* mRNA in the testis of SD donor voles were not observed in F-SD recipient voles. No significant response of serum GnRH levels or *GnRH* gene expression in the hypothalamus and testis were not observed in both donor and recipient voles.

#### Gut microbiota

Beta diversity of gut microbes showed distinction between groups in both donor and recipient voles (Fig. 3b vs Fig. 5b), while only the alpha diversity (Fig. 3a vs Fig. 5a) of F-SD recipient voles was significantly lower than that of F-LD recipient voles. At the genus level, *Oscillibacter* was enriched in both SD and F-SD voles at week 4. For both donor and recipient voles, *Prevotella* was negatively correlated with serum MT levels, but positively correlated with serum FSH levels; *Ruminococcus* was positively correlated with serum FSH levels. Furthermore, in both donor and recipient voles, gut microbes were associated with serum MT, FSH, and LH levels, hypothalamic *Kiss-1* and *GPR54* mRNA, and testicular *Dio2/Dio3* ratio, *Kiss-1*, and *Stra8* expression. Notably, significant correlations between the relative abundance of gut microbes and hormones or genes expression levels in recipient voles were fewer than those in donor voles (Fig. 3c vs Fig. 5c).

## Discussion

Photoperiod is well known to regulate seasonal breeding or aggressive behavior of animals, but the association of the gut microbiota with reproduction and behavior of animals under photoperiod acclimation remains unclear. In this study, we firstly found that gut microbes showed distinct responses to photoperiod treatment, and transplantation of these gut microbes in recipient voles induced similar changes of three hormones (MT, FSH, and LH) and three genes (hypothalamic *Kiss-1*, testicular *Dio3*, and *Dio2/Dio3* ratio) to those in donor voles. Several microbe taxa showed significant association with hormones or gene expression levels. In addition, transplantation of gut microbiota could significantly alter circadian rhythm peaks of recipient voles, but had a weak effect on their circadian behavioral patterns and aggressive behaviors. Our results suggest that gut microbiota is closely associated with reproductive hormones or genes in regulating seasonal breeding and circadian rhythm of Brandt’s voles.

### Effects of photoperiod on reproductive physiology and behavior

There are many behavioral and physiological strategies for small rodents to cope with photoperiod changes in the natural environment. Consistent with the results in hamsters and voles [52–54], we also observed that SD photoperiod decreased the body mass and genital organs of Brandt’s voles, as compared to LD photoperiod. This strategy is often used by voles to reduce energy consumption in cold winter with low temperature and less food resources [55].

Melatonin is a key hormone that triggers adaptation to photoperiod change in most seasonal breeding animals. In this study, we found that SD photoperiod promoted MT secretion and inhibited the secretion of reproductive hormones (FSH, LH, and T) and the expression of hypothalamic genes (*Dio2*, *Kiss-1*, and *Rfrp-3*), which is consistent with several previous studies [2, 56] and our first prediction. These results showed that SD photoperiod inhibited seasonal breeding of animals. Notably, the hypothalamic peptide Kisspeptin and RFRP-3 were regulated by MT, and they were effective modulators of GnRH neuronal activity and had a positive effect on the HPG axis [57]. The opposite function of *Dio2/3* in the testis regulated by thyroid hormone (TH) may alter testicular development and promote spermatogenesis [58]. However, we did not find significant difference in serum GnRH levels, indicating that the GnRH neurons of Brandt’s voles were less sensitive to light, which is consistent with observation of a previous study [2], or may be due to strong negative feedback of T on GnRH [59]. In addition, we found that SD photoperiod inhibited testicular *Kiss-1* and *GPR54* expression, further confirming the observation that testicular Kisspeptin/GPR54 signaling controls steroid production and sperm function [60]. MT binding to its receptor directly affects androgen production of Leydig cells, which affects development of mouse testis [61]. Our data provide evidence that high concentration of MT under SD condition may stimulate expression of *Stra8* (the markers expressing meiotic spermatogonia) in the testis, which then would reduce sperm production and ultimately cause gonad atrophy.

We found SD photoperiod increased the aggressiveness of voles, which was similar to a study in Siberian hamsters [14], and supports our first prediction. Animals tend to increase affinity and to reduce aggression during the breeding season for minimizing harm to litters, while they would compete with each other for food and shelters during the non-breeding season [62]. Besides, we found that photoperiod acclimation altered the circadian rhythms of Brandt’s voles with the highest peak appearing before dark in LD photoperiod, but before dawn in SD photoperiod, this is consistent with a previous study that circadian behavioral rhythms of the voles were regulated by the photoperiod initiation [63].

### Effects of photoperiod on gut microbiota and the relationship between microbiota and hormones or genes expression

Microbial diversity is affected by the photoperiod and could be the result of co-evolution between hosts and microbial community [64]. Alpha diversity of voles from both long-day and short-day photoperiod groups at week 8 was significantly higher than those at weeks 2, 4, and 6. A similar time-dependent pattern was also found in Siberian hamsters [14, 30]. Beta diversity of microbiota diverged between the SD and LD groups at weeks 4, 6, and 8, and the differences in intestinal microbial composition increased along with time supporting our second prediction. In addition, the most abundant phyla in the colon of Brandt’s voles are *Firmicutes* and *Bacteroidetes*, which are involved in food fermentation [65]. The products of food fermentation not only provide energy for the host, but also alter energy metabolism by communicating with the host neuroendocrine system [66]. The ratio of *Firmicutes* to *Bacteroidetes* (F/B) increased under SD photoperiod, and cold acclimation also increased F/B ratio [67], indicating weight loss of Brandt’s voles in winter (i.e., short-day photoperiod and low temperature) may be related to the increase of F/B ratio. At the genus level, SD photoperiod increased the relative abundance of *Ruminococcus* and *Lactobacillus*, whereas decreased the relative abundance of *Alistipes*. Food intake was reduced during SD photoperiod, though this change was not statistically significant, may cause insufficient nutrient supply in Brandt’s voles. As a symbiotic bacterium, *Ruminococcus* degrades and ferments polysaccharides to provide nutrients for the host [68]. Previous studies have shown the proportion of *Alistipes* in obese humans is significantly higher than non-obese [69], which could function to enhance the sugar transport ability of intestinal mucosal cells. This suggests that the increase in body mass of voles caused by LD photoperiod may be related to increase of *Alistipes* [70]. *Lactobacillus* is beneficial to the formation of the body’s immune system and inhibits growth of other harmful bacteria [71]. It was enriched in the SD group and caused an increase in the proportion of *Lactobacillaceae* at the family level. *Lactobacillus* is found in human semen with unexplained infertility and significantly reduces sperm motility [72]. We speculate that reduction in reproduction performance caused by SD photoperiod might be related to increase of *Lactobacillus* in voles.

Previous studies showed that the pineal gland may be responsible for the seasonal changes in gut microbiota and ultimately alters the physiology and behavior of the host in response to seasonal changes [30]. Melatonin supplementation can increase the relative abundance of *Lactobacillus* and improve gastrointestinal health and intestinal microbiome dysregulation in patients with inflammatory bowel disease [33, 34] and benefit intestinal immunity [73]. In this study, *Lactobacillus* was positively correlated with serum MT, supporting the previous observation. FSH and LH levels were found to be significantly associated with a variety of genera, including *Barnesiella*, *Prevotella*, *Lactobacillus*, *Acetatifactor*, *Clostridium_XIVa*, and *Ruminococcus*, probably because sex hormones participate in regulation of bacterial metabolism through steroid receptors and alter the relative abundance of microbiota [74]. In addition, we found there was a significant positive correlation between *Clostridium_XIVa* and testicular *Stra8* mRNA, suggesting that gut microbiota may be involved in the meiosis process of testicular germ cells in male voles. *Kiss-1* and *GPR54* in the hypothalamus and testis were significantly correlated with microorganisms, suggesting that Kisspeptin/GPR54 system in central and peripheral tissues might be key genes in regulating various reproductive and metabolic pathways of microbiota in the host. However, our results were obtained based on associations of gut microbes with reproductive hormones and genes, and further research should be conducted to understand the specific mechanisms operating in these correlations.

### Effects of transplanted gut microbiota on hormones or gene expression and behavior

Although gut microbes have been proved to play a vital role in regulating various physiological processes and behavior of the host [16], their role in regulating photoperiod-related reproduction has never been investigated. The hypothalamus in the brain is the primary location responsible for receiving humoral and neural signals from the gut microbiota [75]. We found that transplantation of gut microbiota-induced similar response of recipient voles to those of donor voles for three hormones (MT, FSH, and LH) and three genes (*Kiss-1* in the hypothalamus, *Dio3* and *Dio2/Dio3* ratio in the testis) (Fig. 6), supporting our third prediction, and providing first evidence that gut microbes are involved in regulation of seasonal breeding animals. However, there were also differences in some gene expression between donor and recipient voles (e.g., serum T of donor voles; *Dio2* and *Rfrp-3* in the hypothalamus of donor voles; *Dio2*, *Kiss*-1, *GRP54*, and *Stra8* in the testis of donor voles; *GRP54* in the hypothalamus of recipient voles). No significant difference of GnRH or *GnRH* gene expression were observed in both donor and recipient voles. Based on our results, we speculate that the SD-exposed microbiota may inhibit the Kisspeptin/GPR54 signaling pathway in the hypothalamus by increasing MT concentration, decreasing FSH and LH secretion by the pituitary gland through co-expression or neuronal connection with GnRH, and ultimately decrease *Dio2/Dio3* ratio and increase *Dio3* mRNA in the testis; and vice versa. Our results suggest that gut microbiota is closely associated with the reproductive genes and hormones in central and peripheral tissues in affecting the seasonal breeding of Brandt’s voles. Gut microbiota also affect deglucuronidation of androgens and intestinal metabolism [76]. But we did not find significant effect of transplanted microbes on serum T level; however, the trend in recipient voles was similar to that in donor voles, likely caused by insufficient sample size. Notably, transplanted microbiota only regulated a partial genes and hormones in recipient voles and had little effect on other physiological indicators (body mass, food intake, and genital organs). It is obvious that the ability of gut microbes to regulate reproduction in the recipient voles is relatively weaker than in donor voles, suggesting they are likely a complementary mechanism to photoperiod in regulating seasonal reproduction.

Many previous studies indicate that gut microbiota is closely associated with behavioral disorders in humans [77], but the effects photoperiod-exposed microbiota on aggressive behavior and daily rhythms in animals remain unclear. Serotonin (5-hydroxytryptamine; 5-HT), a precursor of melatonin (MT), is regulated by photoperiod and negatively correlated with aggressive behavior in rodents [78, 79]. Intestinal enterochromaffin cells produce intestinal serotonin, which is stored and obtained by circulating platelets, participates in regulation of the membrane permeability in the brain, intestine, and other organs [80]. The intestinal serotonin may play a vital role in the whole body including the brain as a continuous regulatory signal similar to a hormone [80]. Increasing evidence suggests that neurotransmitters synthesized or released by gut microbiota play a crucial role in regulating sleep-wake rhythms [81]. Some bacteria, for example, the genus *Bacillus* synthesizes gamma-aminobutyric acid (GABA), whereas bacteria of *Streptococcus* or *Escherichia* synthesize 5-HT [82]. These hormones and neurotransmitters responsible for regulating daily rhythms were influenced by gut microbiota [83]. In SD donor voles, increased aggressive behavior was positively associated with an increase in MT, which is consistent with previous observations and supports our first prediction. Transplantation of gut microbes significantly altered MT in F-SD recipient voles, but only induced slightly increase of aggression, likely due to insufficient sample size, partially supporting our third prediction. Both LD donor and recipient voles showed similar difference in circadian rhythm peaks, but circadian rhythm peak of F-SD recipient voles was different from those of both LD donor and recipient voles, indicating transplantation of gut microbes could disrupt circadian rhythms of recipient voles.

### Effects of fecal transplanted microbiota on gut microbiota of recipient voles and its association with hormones or gene expression

Unlike donor voles, alpha diversity of recipient voles differed significantly between the F-LD and F-SD groups. Changes in beta diversity of recipient voles were consistent with those of donor voles. At the phylum, family, and genus levels, the relative abundance of microbes between recipient and donor voles was very large, suggesting that the gut microbiota of voles heavily depended upon the host’s environment. SD-exposed microbiota at week 8 increased the relative abundance of *Verrucomicrobia*, *Verrucomicrobiaceae*, and *Akkermansia* in recipient voles, but no such difference was found in donor voles. *Akkermansia* is known to improve glucose/lipid metabolism, prolongating the lifespan of healthy rodents, and inducing production of the anti-inflammatory cytokine interleukin 10 (IL-10) [84–87]. Thus, high relative abundance of *Akkermansia* in F-SD voles may benefit survival of voles in non-breeding seasons with harsh environment.

The interaction between sex hormones and gut microbiota is often reciprocal [25]. Similar to donor voles, *Prevotella* and *Ruminococcus* of recipient voles were significantly correlated to reproductive hormones (e.g., MT and FSH), indicating that these two genera were key bacteria conducive to reproduction of rodents. A recent study found that *Prevotella* is present in Human Papilloma Virus (HPV)-positive semen that causes male infertility, which may negatively affect sperm motility [72]. In recipient voles, hypothalamic *Kiss-1* and *GPR54* were associated with microorganisms (e.g., *Barnesiella*, *Prevotella*, and *Clostridium_XIVa*), suggesting these gut microbes may regulate reproduction through the Kisspeptin/GPR54 pathway in the hypothalamus.

### Difference between the control and FMT groups

Control groups are important to infer the effects of FMT groups on recipient voles. The Con and Ab groups were exposed to LD photoperiod without or with pre-antibiotic treatment, respectively. We found the Ab group had much more significant different response in hormones and genes with the Con group (*n* = 10) than with the F-LD group (*n* = 4) (Table S12), indicating pre-antibiotic treatment was better for inferring the effects of FMT; results of the Con group without pre-antibiotic treatment were hard to explain the changes in the FMT groups with pre-antibiotic treatment. As expected, because the Ab and F-LD group were exposed to LD photoperiod or FMT from LD donor voles, and were both treated with antibiotic, it is reasonable that their responses were very similar to each other, except for four hormones (MT, FSH, LH, and T; Table S12). This difference was likely caused by different gut microbes or their metabolites between the Ab and FMT groups representing different exposure environments (Figure S5f). As expected, the F-SD group exposed to FMT from SD donor voles showed a large difference with Ab group with 7 hormones or genes; in contrast, the F-LD group showed a different response in only 4 hormones with Ab group, supporting the observed different effects between the F-LD and F-SD groups. Due to difference in photoperiod and pre-antibiotic treatment, the difference in hormones and genes (*n* = 8) was also large between the F-SD and Con groups.

Our results also indicated that the circadian rhythm and gut microbiota showed significant difference between the control and the FMT groups. In particular, gut microbiota of Con group showed more distinction in beta diversity from the other three groups, which might be caused by the difference in pre-antibiotic treatment because antibiotic could significantly alter the gut microbes (see below). The difference in aggressive behavior or circadian rhythm activity was small among the four groups, which was consistent with the observation that there was no significant difference in aggression intensity or small difference in locomotion activity between the F-SD and F-LD groups.

Antibiotics are known to modulate the structure and composition of the gut microbiota [88]. Antibiotic pre-treatment prior to FMT is widely used for efficient colonization of the donor microbiota into the recipient [89]. However, its effects on treatment group are less investigated. We found that antibiotic pre-treatment reduced microbial community diversity and altered its composition and structure of recipient voles, which is consistent with a study on Mongolian gerbils (*Meriones unguiculatus*) [90]. Antibiotic pre-treatment significantly increased colon length in mice, suggesting that antibiotics may alter physiological changes in rodents [89]. In this study, as compared to control groups, hormone (serum FSH levels) and genes (hypothalamic *GPR54* mRNA and testicular *Dio2* mRNA and *Dio2/Dio3* ratio) in the central and peripheral tissues were significantly elevated in Ab voles and may result in higher values of reproductive performance in F-SD and F-LD voles than the control group. Therefore, direct comparison in physiological responses between the F-SD and F-LD groups would be clearer.

### Limitation of this study

This study suffered several limitations. First, our results were obtained based on correlational data; thus, we were not able to distinguish the mechanisms by which gut microbiota and their metabolites affect reproductive hormones and genes. They may interact with each other in regulating the seasonal breeding animals. Future studies should be directed to examine the distinct role of microbial communities and its interaction with hormones or genes on physiological response to photoperiod. Second, voles of SD group at week 8 showed a significantly higher attack intensity than voles of LD group, which is consistent with previous studies (e.g., Siberian hamster) [14], but the underlying mechanisms remain unclear [91]. Previous studies suggest that aggression may increase serum corticosterone levels [38] and alter the relative abundance of gut microbiota [45], which may have an indistinguishable effect with photoperiod on hormone, genes, and gut microbiota. However, we found there was no significant association of number of attacks and attack duration with levels of all hormones and expression of all genes (except for testicular *GnRH*) in both SD and LD groups (Table S10), indicating the effect of attack intensity would be minor in our study. Future studies should be directed to assess the impacts of photoperiod-induced aggressive behavior on physiological response to photoperiod.

### Implications

Our study indicated that gut microbiota is associated with photoperiod-induced seasonal breeding, aggression, and circadian rhythm behaviors of animals, which would have significant implications in pest control, livestock animal breeding, and human health management. For example, specific SD-exposed microbiota could be used for improving sleep issues in humans or help to adjust jet-lag of people who travel frequently. Specific SD-exposed microbiota could help to prohibit the reproduction of rodents so as to reduce their overabundant populations. The reproduction of many livestock animals is also regulated by LD photoperiod (e.g., horse and donkey) or SD photoperiod (sheep and camel). Thus, administration of specific LD- or SD-exposed microbiota could benefit breeding of these livestock animals. Further studies are needed to identify the key microbes and to figure out the underlying mechanisms of gut microbiota in regulating seasonal reproduction and behaviors of animals.

## Conclusions

Our study firstly reveals that gut microbiota is involved in photoperiodic regulation of seasonal breeding, aggressive behavior, and daily rhythms in animals. Gut microbiota is sensitive to photoperiod change, and transplantation of SD- or LD-exposed microbiota can cause similar change of reproductive hormone or genes, and aggressive behavior or daily rhythms in recipient voles, as compared to donor voles. Gut microbiota may regulate the seasonal reproduction, aggressive behavior, or daily rhythms though HPG axis, melatonin, and Kisspeptin/GPR54 system. Taken together, our results provide novel insight into the interaction between host and microbes in regulating seasonal reproduction and behavior, and significant cues in managing animal breeding and human health.

## Supplementary Information

**Additional file 1: Figure S1.** Diversity and composition of gut microbiota in the LD and SD groups of Brandt’s voles.

**Additional file 2: Figure S2.** FMT alters reproductive genes, genital organs, and bacterial diversity and composition of Brandt’s voles.

**Additional file 3: Figure S3.** Differences in body mass, food intake, and behavior patterns between the control groups and the fecal microbiota transplantation (FMT) groups.

**Additional file 4: Figure S4.** Differences in hormone, hypothalamic and testicular gene, and genital organ between the control groups and the FMT groups.

**Additional file 5: Figure S5.** Differences in diversity and composition of microbiota between the control groups and the FMT groups.

**Additional file 6: Table S1.** Information on gene primers of Brandt's voles.

**Additional file 7: Table S2.** Spearman correlations between ASVs and serum hormones levels in the photoperiod experiment.

**Additional file 8: Table S3.** Spearman correlations between ASVs and hypothalamic genes in the photoperiod experiment.

**Additional file 9: Table S4.** Spearman correlation coefficients (*r*) between ASVs and testicular genes in the photoperiod experiment.

**Additional file 10: Table S5.** Spearman correlations significance (*P*-value) between ASVs and testicular genes in the photoperiod experiment.

**Additional file 11: Table S6.** Spearman correlations between ASVs and serum hormones levels in the FMT experiment.

**Additional file 12: Table S7.** Spearman correlations between ASVs and hypothalamic genes in the FMT experiment.

**Additional file 13: Table S8.** Spearman correlation coefficients (*r*) between ASVs and testicular genes in the FMT experiment.

**Additional file 14: Table S9.** Spearman correlations significance (*P*-value) between ASVs and testicular genes in the FMT experiment.

**Additional file 15: Table S10.** Spearman correlations of aggressive behavior with hormones and genes in the photoperiod experiment.

**Additional file 16: Table S11.** Difference in physiological indices between the Con and Ab groups, Con and F-LD groups, Con and F-SD groups, Ab and F-LD groups, Ab and F-SD groups, and F-LD and F-SD groups in the FMT experiment.

**Additional file 17: Table S12.** Difference in physiological indices of gut microbiota between the Con and F-LD groups, Con and F-SD groups, Ab and F-LD groups, and Ab and F-SD groups in the FMT experiment.

**Additional file 18: Table S13.** Difference in alpha diversity of gut microbiota between the Con and Ab groups, Con and F-LD groups, Con and F-SD groups, Ab and F-LD groups, Ab and F-SD groups, and F-LD and F-SD groups in the FMT experiment.

**Additional file 19: Table S14.** Difference in beta diversity of gut microbiota between the Con and Ab groups, Con and F-LD groups, Con and F-SD groups, Ab and F-LD groups, Ab and F-SD groups, and F-LD and F-SD groups in the FMT experiment.

**Additional file 20.** Photoperiod experiment and FMT experiment_data.

**Additional file 21.** Rcode.R.

## Data Availability

The 16S rRNA gene sequencing data is available in the Science Data Bank repository 10.11922/sciencedb.o00019.00006 and 10.11922/sciencedb.o00019.00008. All data can be obtained in this manuscript or from the authors upon request.

## References

[1] Guh YJ, Tamai TK, Yoshimura T (2019). The underlying mechanisms of vertebrate seasonal reproduction. Proc Jpn Acad Ser B Phys Biol Sci.

[2] Wang D, Li N, Tian L, Ren F, Li Z, Chen Y (2019). Dynamic expressions of hypothalamic genes regulate seasonal breeding in a natural rodent population. Mol Ecol.

[3] Tavolaro FM, Thomson LM, Ross AW, Morgan PJ, Helfer G (2015). Photoperiodic effects on seasonal physiology, reproductive status and hypothalamic gene expression in young male F344 rats. J Neuroendocrinol.

[4] Lee BH, Bussi IL, De La Iglesia HO, Hague C, Koh DS, Hille B (2020). Two indoleamines are secreted from rat pineal gland at night and act on melatonin receptors but are not night hormones. J Pineal Res.

[5] Revel FG, Masson-Pévet M, Pévet P, Mikkelsen JD, Simonneaux V (2009). Melatonin controls seasonal breeding by a network of hypothalamic targets. Neuroendocrinology..

[6] Boczek-Leszczyk E, Stempniak B, Juszczak M (2010). Vasopressin release from the rat hypothalamo-neurohypophysial system: effects of gonadotrophin-releasing hormone (GnRH), its analogues and melatonin. J Physiol Pharmacol.

[7] Ono H, Hoshino Y, Yasuo S, Watanabe M, Nakane Y, Murai A (2008). Involvement of thyrotropin in photoperiodic signal transduction in mice. Proc Natl Acad Sci U S A.

[8] Liu L, Chen Y, Wang D, Li N, Guo C, Liu X (2018). Cloning and expression characterization in hypothalamic *Dio2/3* under a natural photoperiod in the domesticated Brandt's vole (*Lasiopodomys brandtii*). Gen Comp Endocrinol.

[9] Bailey AM, Legan SJ, Demas GE (2017). Exogenous kisspeptin enhances seasonal reproductive function in male Siberian hamsters. Funct Ecol.

[10] Ubuka T, Inoue K, Fukuda Y, Mizuno T, Ukena K, Kriegsfeld LJ (2012). Identification, expression, and physiological functions of Siberian hamster gonadotropin-inhibitory hormone. Endocrinology..

[11] Zhou Q, Li Y, Nie R, Friel P, Mitchell D, Evanoff RM (2008). Expression of stimulated by retinoic acid gene 8 (*Stra8*) and maturation of murine gonocytes and spermatogonia induced by retinoic acid in vitro. Biol Reprod.

[12] Luo J, Yang Y, Ji X, He W, Fan J, Huang Y (2021). NGF rescues spermatogenesis in azoospermic mice. Reprod Sci.

[13] Soma KK (2006). Testosterone and aggression: Berthold, birds and beyond. J Neuroendocrinol.

[14] Ren CC, Sylvia KE, Munley KM, Deyoe JE, Henderson SG, Vu MP (2020). Photoperiod modulates the gut microbiome and aggressive behavior in Siberian hamsters. J Exp Biol.

[15] Shreiner AB, Kao JY, Young VB (2015). The gut microbiome in health and in disease. Curr Opin Gastroenterol.

[16] Foster JA, Lyte M, Meyer E, Cryan JF (2016). Gut microbiota and brain function: an evolving field in neuroscience. Int J Neuropsychopharmacol.

[17] Xiong W (2022). Intestinal microbiota in various animals. Integr Zool.

[18] Insenser M, Murri M, Del Campo R, Martínez-García M, Fernández-Durán E, Escobar-Morreale HF (2018). Gut microbiota and the polycystic ovary syndrome: influence of sex, sex hormones, and obesity. J Clin Endocrinol Metab.

[19] Wang B, Zhang L, Zhu SW, Zhang JD, Duan LP (2019). Short chain fatty acids contribute to gut microbiota-induced promotion of colonic melatonin receptor expression. J Biol Regul Homeost Agents.

[20] Yin J, Li Y, Han H, Ma J, Yin Y (2020). Administration of exogenous melatonin improves the diurnal rhythms of the gut microbiota in mice fed a high-fat diet. mSystems..

[21] Zhang P, Feng Y, Li L, Ge W, Yu S, Hao Y (2021). Improvement in sperm quality and spermatogenesis following faecal microbiota transplantation from alginate oligosaccharide dosed mice. Gut..

[22] Cryan JF, O'mahony SM. (2011). The microbiome-gut-brain axis: from bowel to behavior. Neurogastroenterol Motil.

[23] Yan L, Tang L, Zhou Z, Lu W, Wang B, Sun Z (2022). Metagenomics reveals contrasting energy utilization efficiencies of captive and wild camels (*Camelus ferus*). Integr Zool.

[24] Lundy SD, Sangwan N, Parekh NV, Selvam MKP, Gupta S, Mccaffrey P (2021). Functional and taxonomic dysbiosis of the gut, urine, and semen microbiomes in male infertility. Eur Urol.

[25] Neuman H, Debelius JW, Knight R, Koren O (2015). Microbial endocrinology: the interplay between the microbiota and the endocrine system. FEMS Microbiol Rev.

[26] Xu X, Zhang Z (2021). Sex- and age-specific variation of gut microbiota in Brandt's voles. PeerJ..

[27] Liu J, Huang S, Li G, Zhao J, Lu W, Zhang Z (2020). High housing density increases stress hormone- or disease-associated fecal microbiota in male Brandt's voles (*Lasiopodomys brandtii*). Horm Behav.

[28] Li G, Li J, Kohl KD, Yin B, Wei W, Wan X (2019). Dietary shifts influenced by livestock grazing shape the gut microbiota composition and co-occurrence networks in a local rodent species. J Anim Ecol.

[29] Amato KR, Yeoman CJ, Kent A, Righini N, Carbonero F, Estrada A (2013). Habitat degradation impacts black howler monkey (*Alouatta pigra*) gastrointestinal microbiomes. ISME J.

[30] Shor EK, Brown SP, Freeman DA (2020). A novel role for the pineal gland: regulating seasonal shifts in the gut microbiota of Siberian hamsters. J Pineal Res.

[31] Chakravarti S, Rizvi S (2008). Physiological effects of melatonin: implication on human health. Biomedicine..

[32] Mao C, Xu Y, Shi L, Guo S, Jin X, Yan S (2019). Effects of photoperiod change on melatonin secretion, immune function and antioxidant status of cashmere goats. Animals (Basel).

[33] Kim SW, Kim S, Son M, Cheon JH, Park YS (2020). Melatonin controls microbiota in colitis by goblet cell differentiation and antimicrobial peptide production through Toll-like receptor 4 signalling. Sci Rep.

[34] Gao T, Wang Z, Dong Y, Cao J, Lin R, Wang X (2019). Role of melatonin in sleep deprivation-induced intestinal barrier dysfunction in mice. J Pineal Res.

[35] Li G, Yin B, Wan X, Wei W, Wang G, Krebs CJ (2016). Successive sheep grazing reduces population density of Brandt’s voles in steppe grassland by altering food resources: a large manipulative experiment. Oecologia..

[36] Zhong Z, Li G, Sanders D, Wang D, Holt R, Zhang Z (2022). A rodent herbivore reduces its predation risk through ecosystem engineering. Curr Biol.

[37] Huang S, Li G, Pan Y, Song M, Zhao J, Wan X (2020). Density-induced social stress alters oxytocin and vasopressin activities in the brain of a small rodent species. Integr Zool.

[38] Huang S, Li G, Pan Y, Liu J, Zhao J, Zhang X (2021). Population variation alters aggression-associated oxytocin and vasopressin expressions in brains of Brandt’s voles in field conditions. Front Zool.

[39] Li G, Wan X, Yin B, Wei W, Hou X, Zhang X (2021). Timing outweighs magnitude of rainfall in shaping population dynamics of a small mammal species in steppe grassland. Proc Natl Acad Sci U S A.

[40] Cui C, Xie Y, Hua Y, Yang S, Yin B, Wei W (2020). Brandt’s vole (*Lasiopodomys brandtii*) affects its habitat quality by altering plant community composition. Biologia.

[41] Markle JG, Frank DN, Mortin-Toth S, Robertson CE, Feazel LM, Rolle-Kampczyk U (2013). Sex differences in the gut microbiome drive hormone-dependent regulation of autoimmunity. Science..

[42] Van Rosmalen L, Van Dalum J, Hazlerigg DG, Hut RA (2020). Gonads or body? Differences in gonadal and somatic photoperiodic growth response in two vole species. J Exp Biol.

[43] Xia Q, Di R, He XY, Wei CH, Chu MX (2020). Expression analysis of *DIO2*, *EYA3*, *KISS1* and *GPR54* genes in year-round estrous and seasonally estrous rams. Arch Anim Breed.

[44] Takahashi A, Miczek KA (2014). Neurogenetics of aggressive behavior: studies in rodents. Curr Top Behav Neurosci.

[45] Zhao J, Li G, Lu W, Huang S, Zhang Z (2020). Dominant and subordinate relationship formed by repeated social encounters alters gut microbiota in greater long-tailed hamsters. Microb Ecol.

[46] Chen Y, Liu L, Li Z, Wang D, Li N, Song Y (2017). Molecular cloning and characterization of kiss1 in Brandt's voles (*Lasiopodomys brandtii*). Comp Biochem Physiol B.

[47] Liu L, Chen Y, Li N, Wang D, Ren F, Song Y (2017). Molecular cloning, tissue distribution and expression of *GnRH* gene in different developmental stages of *Lasiopodomys brandtii*. J Zool.

[48] Zhang X, Sukhchuluun G, Bo T, Chi Q, Yang J, Chen B (2018). Huddling remodels gut microbiota to reduce energy requirements in a small mammal species during cold exposure. Microbiome..

[49] Edgar RC, Flyvbjerg H (2015). Error filtering, pair assembly and error correction for next-generation sequencing reads. Bioinformatics..

[50] Eckburg PB, Bik EM, Bernstein CN, Purdom E, Dethlefsen L, Sargent M (2005). Diversity of the human intestinal microbial flora. Science..

[51] Li G, Yin B, Li J, Wang J, Zhang Z (2020). Host-microbiota interaction helps to explain the bottom-up effects of climate change on a small rodent species. ISME J.

[52] Li X, Wang D (2007). Photoperiod and temperature can regulate body mass, serum leptin concentration, and uncoupling protein 1 in Brandt's voles (*Lasiopodomys brandtii*) and Mongolian gerbils (*Meriones unguiculatus*). Physiol Biochem Zool.

[53] Dai X, Zhou L, Xu T, Wang Q, Luo B, Li Y (2020). Reproductive responses of the male Brandt’s vole, *Lasiopodomys brandtii* (Rodentia: Cricetidae) to tannic acid. Zoologia..

[54] Haugg E, Herwig A, Diedrich V (2021). Body temperature and activity adaptation of short photoperiod-exposed djungarian hamsters (*Phodopus sungorus*): timing, traits, and torpor. Front Physiol.

[55] Aars J, Ims RA (2002). Intrinsic and climatic determinants of population demography: the winter dynamics of tundra voles. Ecology..

[56] Revel FG, Saboureau M, Masson-Pévet M, Pévet P, Mikkelsen JD, Simonneaux V (2006). Kisspeptin mediates the photoperiodic control of reproduction in hamsters. Curr Biol.

[57] Simonneaux V, Ancel C, Gauer F, Poirel V-J (2013). Kisspeptins and RFRP-3 act in concert to synchronize rodent reproduction with seasons. Front Neurosci.

[58] Hernandez A, Martinez ME (2020). Thyroid hormone action in the developing testis: intergenerational epigenetics. J Endocrinol.

[59] Tilbrook AJ, Clarke IJ (2001). Negative feedback regulation of the secretion and ations of gonadotropin-releasing hormone in males. Biol Reprod.

[60] Sharma A, Thaventhiran T, Minhas S, Dhillo WS, Jayasena CN (2020). Kisspeptin and testicular function-is it necessary?. Int J Mol Sci.

[61] Kasahara T, Abe K, Mekada K, Yoshiki A, Kato T (2010). Genetic variation of melatonin productivity in laboratory mice under domestication. Proc Natl Acad Sci U S A.

[62] Bailey AM, Rendon NM, O'malley KJ, Demas GE (2016). Food as a supplementary cue triggers seasonal changes in aggression, but not reproduction, in Siberian hamsters. Physiol Behav.

[63] Sun H, Li C, Zhang Y, Jiang M, Dong Q, Wang Z (2020). Light-resetting impact on behavior and the central circadian clock in two vole species (genus: Lasiopodomys). Comp BiochemI Phys B.

[64] Ley RE, Hamady M, Lozupone C, Turnbaugh PJ, Ramey RR, Bircher JS (2008). Evolution of mammals and their gut microbes. Science..

[65] Ottman N, Smidt H, De Vos WM, Belzer C (2012). The function of our microbiota: who is out there and what do they do?. Front Cell Infect Mi.

[66] Ramakrishna BS (2013). Role of the gut microbiota in human nutrition and metabolism. J Gastroenterol Hepatol.

[67] Bo T, Zhang X, Wen J, Deng K, Qin X, Wang D (2019). The microbiota–gut–brain interaction in regulating host metabolic adaptation to cold in male Brandt’s voles (*Lasiopodomys brandtii*). ISME J.

[68] Leschine SB (1995). Cellulose degradation in anaerobic environments. Annu Rev Microbiol.

[69] Clarke SF, Murphy EF, O'sullivan O, Ross RP, O'toole PW, Shanahan F (2013). Targeting the microbiota to address diet-induced obesity: a time dependent challenge. PLoS One.

[70] Agans R, Gordon A, Kramer DL, Perez-Burillo S, Rufián-Henares JA, Paliy O (2018). Dietary fatty acids sustain the growth of the human gut microbiota. Appl Environ Microbiol.

[71] Schillinger U (1989). Antibacterial activity of *Lactobacillus* sake isolated from meat. Appl Environ Microbiol.

[72] Campisciano G, Iebba V, Zito G, Luppi S, Martinelli M, Fischer L (2020). *Lactobacillus iners* and *gasseri*, *Prevotella bivia* and HPV belong to the microbiological signature negatively affecting human reproduction. Microorganisms..

[73] Wang B, Zhu S, Liu Z, Wei H, Zhang L, He M (2020). Increased expression of colonic mucosal melatonin in patients with irritable bowel syndrome correlated with gut dysbiosis. Genom Proteom Bioinf.

[74] Yoon K, Kim N (2021). Roles of sex hormones and gender in the gut microbiota. J Neurogastroenterol Motil.

[75] De Palma G, Blennerhassett P, Lu J, Deng Y, Park AJ, Green W (2015). Microbiota and host determinants of behavioural phenotype in maternally separated mice. Nat Commun.

[76] Colldén H, Landin A, Wallenius V, Elebring E, Fändriks L, Nilsson ME (2019). The gut microbiota is a major regulator of androgen metabolism in intestinal contents. Am J Physiol Endocrinol Metab.

[77] Huang T, Lai J, Du Y, Xu Y, Ruan L, Hu S (2019). Current understanding of gut microbiota in mood disorders: an update of human studies. Front Genet.

[78] Bazhenova EY, Fursenko DV, Kulikova EA, Khotskin NV, Sinyakova NA, Kulikov AA (2019). Effect of photoperiodic alterations on depression-like behavior and the brain serotonin system in mice genetically different in tryptophan hydroxylase 2 activity. Neurosci Lett.

[79] Linnoila VM, Virkkunen M (1992). Aggression, suicidality, and serotonin. J Clin Psychiatry.

[80] Szőke H, Kovács Z, Bókkon I, Vagedes J, Szabó AE, Hegyi G (2020). Gut dysbiosis and serotonin: intestinal 5-HT as a ubiquitous membrane permeability regulator in host tissues, organs, and the brain. Rev Neurosci.

[81] Wagner-Skacel J, Dalkner N, Moerkl S, Kreuzer K, Farzi A, Lackner S (2020). Sleep and microbiome in psychiatric diseases. Nutrients..

[82] Sgro M, Kodila ZN, Brady RD, Reichelt AC, Mychaisuk R, Yamakawa GR. Synchronizing our clocks as we age: the influence of the brain-gut-immune axis on the sleep-wake cycle across the lifespan. Sleep. 2021;45:zsab268. 10.1093/sleep/zsab268 34757429

[83] Ogawa Y, Miyoshi C, Obana N, Yajima K, Hotta-Hirashima N, Ikkyu A (2020). Gut microbiota depletion by chronic antibiotic treatment alters the sleep/wake architecture and sleep EEG power spectra in mice. Sci Rep.

[84] Cani PD, De Vos WM (2017). Next-generation beneficial microbes: the case of *Akkermansia muciniphila*. Front Microbiol.

[85] Plovier H, Everard A, Druart C, Depommier C, Van Hul M, Geurts L (2017). A purified membrane protein from *Akkermansia muciniphila* or the pasteurized bacterium improves metabolism in obese and diabetic mice. Nat Med.

[86] Everard A, Belzer C, Geurts L, Ouwerkerk JP, Druart C, Bindels LB (2013). Cross-talk between *Akkermansia muciniphila* and intestinal epithelium controls diet-induced obesity. Proc Natl Acad Sci U S A.

[87] Shin J, Noh J-R, Choe D, Lee N, Song Y, Cho S (2021). Ageing and rejuvenation models reveal changes in key microbial communities associated with healthy ageing. Microbiome..

[88] Ianiro G, Tilg H, Gasbarrini A (2016). Antibiotics as deep modulators of gut microbiota: between good and evil. Gut..

[89] Freitag TL, Hartikainen A, Jouhten H, Sahl C, Meri S, Anttila V-J (2019). Minor effect of antibiotic pre-treatment on the engraftment of donor microbiota in fecal transplantation in mice. Front Microbiol.

[90] Khakisahneh S, Zhang X, Nouri Z, Wang D (2020). Gut microbiota and host thermoregulation in response to ambient temperature fluctuations. mSystems..

[91] Silva AL, Fry WH, Sweeney C, Trainor BC (2010). Effects of photoperiod and experience on aggressive behavior in female California mice. Behav Brain Res.

