# Supplementary material for: Gut microbiota is associated with the effect of photoperiod on seasonal breeding in male Brandt’s voles (Lasiopodomys brandtii)
Source: Microbiome. 2022 Nov 15;10:194. doi: 10.1186/s40168-022-01381-1 (PMC9664686; doi:10.1186/s40168-022-01381-1)
Supplement: Supplementary file 2 — Additional file 1: Figure S1. Diversity and composition of gut microbiota in the LD and SD groups of Brandt’s voles. [file 40168_2022_1381_MOESM1_ESM.docx]

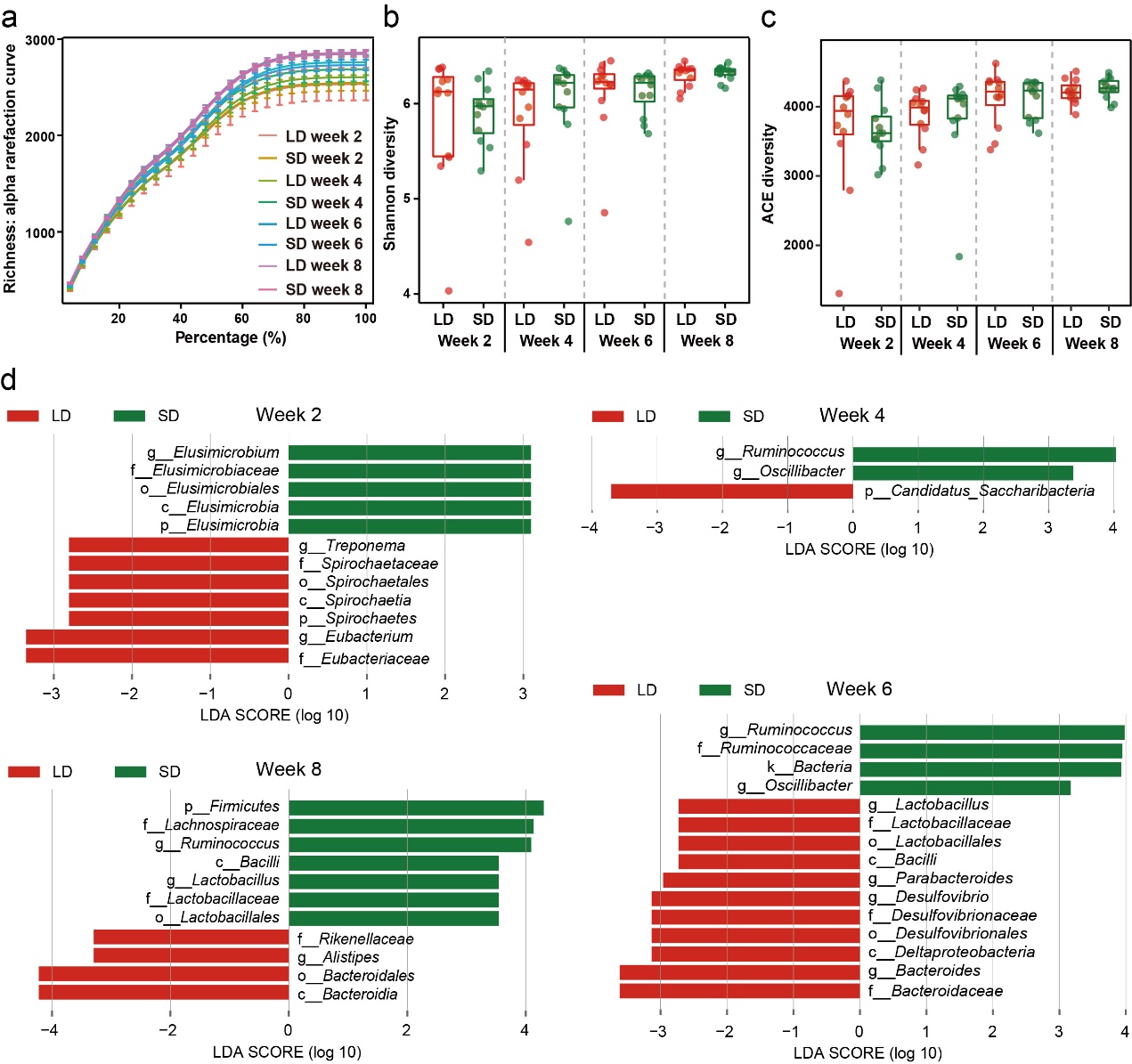


**Figure S1 Diversity and composition of gut microbiota in the LD and SD groups of Brandt’s voles. a** Rarefaction curves of Richness diversity. **b, c** Alpha diversity (Shannon and ACE diversity) of bacterial communities across the LD and SD groups (LMM). **d** The differentially abundant taxa enriched in microbial communities from the LD and SD groups at week 2, 4, 6, and 8 by LEfSe (LDA >2, a < 0.05). LD: long-day photoperiod (16L: 8D); SD: short-day photoperiod (8L: 16D).
