# Supplementary material for: Gut microbiota is associated with the effect of photoperiod on seasonal breeding in male Brandt’s voles (Lasiopodomys brandtii)
Source: Microbiome. 2022 Nov 15;10:194. doi: 10.1186/s40168-022-01381-1 (PMC9664686; doi:10.1186/s40168-022-01381-1)
Supplement: Supplementary file 3 — Additional file 2: Figure S2. FMT alters reproductive genes, genital organs, and bacterial diversity and composition of Brandt’s voles. [file 40168_2022_1381_MOESM2_ESM.docx]

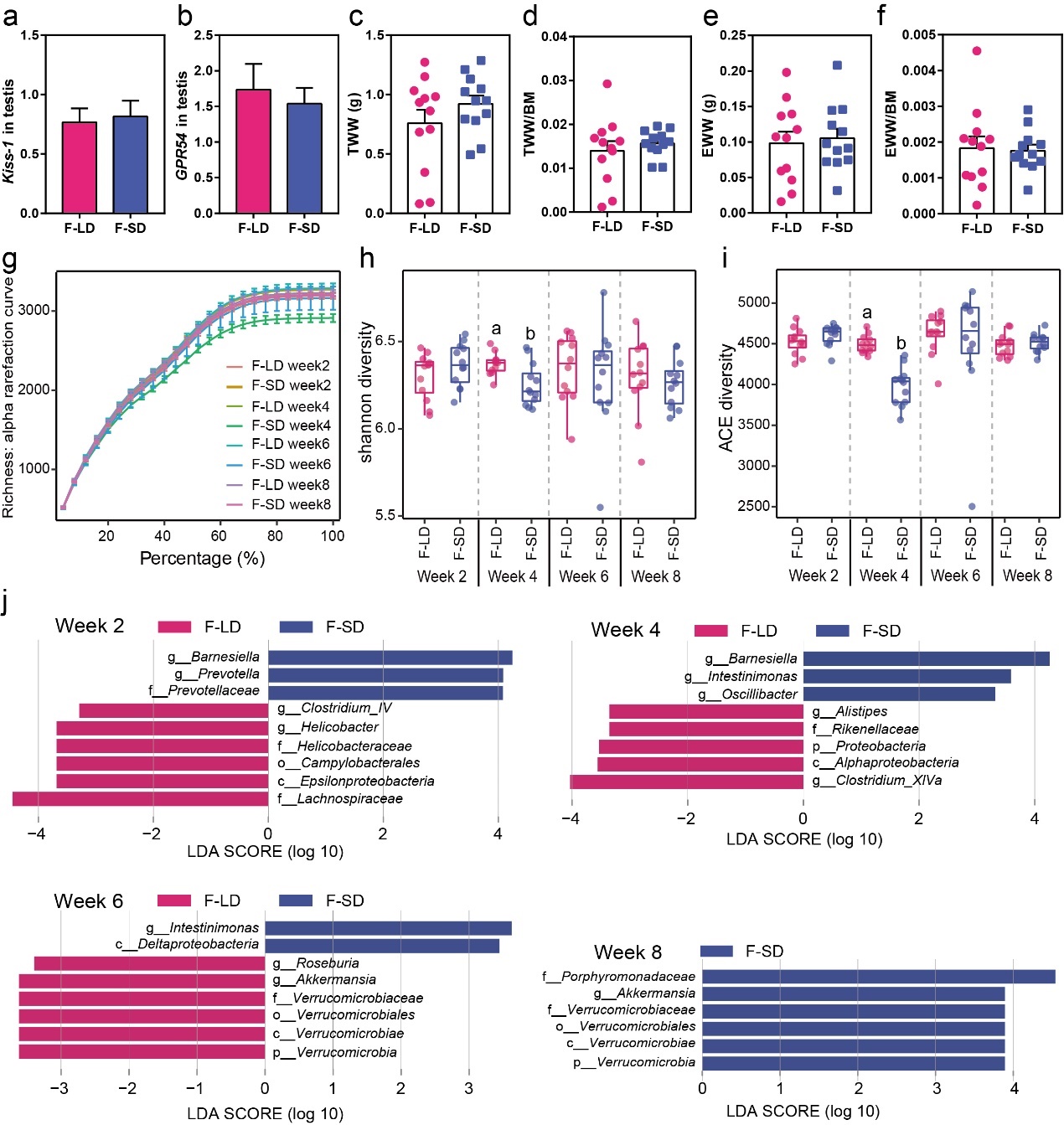


**Figure S2 FMT alters reproductive genes, genital organs, and bacterial diversity and composition of Brandt’s voles.** **a, b** The expression of *Kiss-1* and *GPR54* in testis. **c-f** TWW: testicular wet weight; TWW/BM: the ratio of testicular wet weight to body mass; EWW: epididymis wet weight; EWW/BM: the ratio of epididymis weight to body mass. **g** Rarefaction curves of Richness diversity. **h, i** Comparison of Shannon diversity and ACE diversity of bacterial communities in the F-LD and F-SD groups (means ± SEM). **j** The differentially abundant taxa enriched in microbial communities from the F-LD and F-SD groups at week 2, 4, 6, and 8 by LEfSe (LDA >2, a < 0.05). F-LD: recipients with LD-exposed microbiota; F-SD: recipients with SD-exposed microbiota.
