# Supplementary material for: Gut microbiota is associated with the effect of photoperiod on seasonal breeding in male Brandt’s voles (Lasiopodomys brandtii)
Source: Microbiome. 2022 Nov 15;10:194. doi: 10.1186/s40168-022-01381-1 (PMC9664686; doi:10.1186/s40168-022-01381-1)
Supplement: Supplementary file 4 — Additional file 3: Figure S3. Differences in body mass, food intake, and behavior patterns between the control groups and the fecal microbiota transplantation (FMT) groups. [file 40168_2022_1381_MOESM3_ESM.docx]

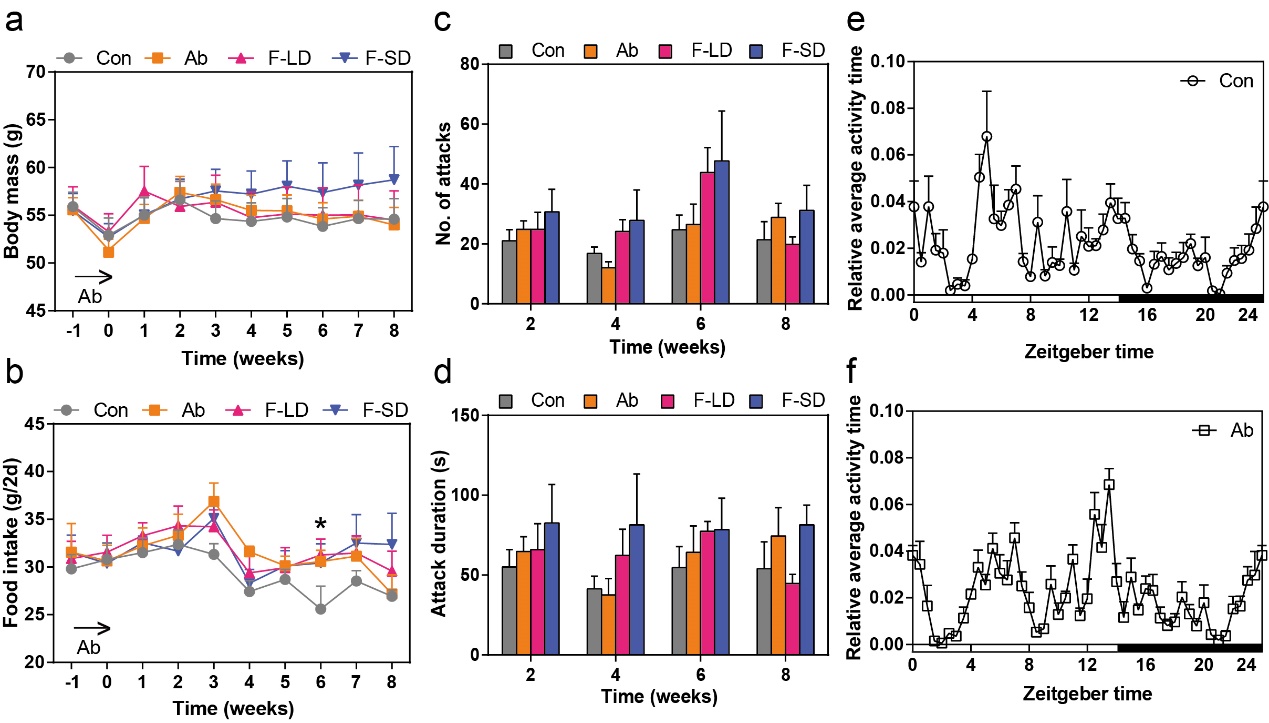


**Figure** **S3** **Differences in body mass, food intake, and behavior patterns between the control groups and the fecal microbiota transplantation (FMT) groups. a, b** Changes in body mass and food intake during the period of all groups. **c, d** Number of attacks and attack duration at week 2, week 4, week 6, and week 8. **e, f** Relative average activity time of voles in 24 hours at the end of saline or antibiotic treatment. Data are means ± SEM
