# Supplementary material for: Gut microbiota is associated with the effect of photoperiod on seasonal breeding in male Brandt’s voles (Lasiopodomys brandtii)
Source: Microbiome. 2022 Nov 15;10:194. doi: 10.1186/s40168-022-01381-1 (PMC9664686; doi:10.1186/s40168-022-01381-1)
Supplement: Supplementary file 5 — Additional file 4: Figure S4. Differences in hormone, hypothalamic and testicular gene, and genital organ between the control groups and the FMT groups. [file 40168_2022_1381_MOESM4_ESM.docx]

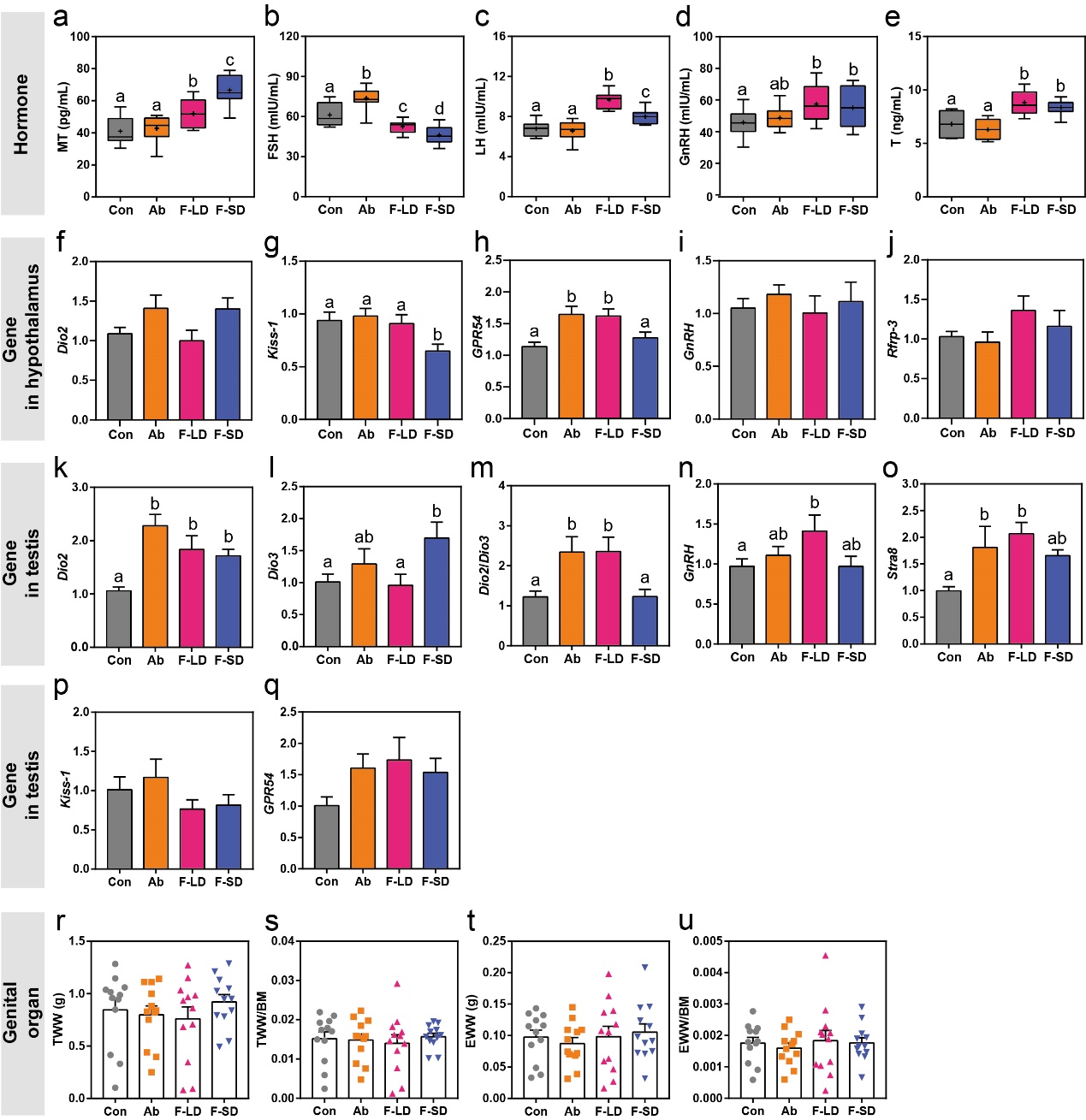


**Figure S4 Differences in hormone, hypothalamic and testicular gene, and genital organ between the control groups and the FMT groups. a-e** MT, FSH, LH, GnRH, and T levels in serum. **f-j** The expression of *Dio2*, *Kiss-1*, *GPR54*, *GnRH*, and *Rfrp-3* in the hypothalamus. **k-q** The expression of *Dio2*, *Dio3*, *Dio2/Dio3* (the ratio of *Dio2* to *Dio3* expression), *GnRH*, *Stra8*, *Kiss-1*, and *GPR54* in the testis. **r** TWW: testicular wet weight. **s** TWW/BM: the ratio of testicular wet weight to body mass. **t** EWW: epididymis wet weight. **u** EWW/BM: the ratio of epididymis weight to body mass. Data are presented as mean ± SEM
