# Supplementary material for: Gut microbiota is associated with the effect of photoperiod on seasonal breeding in male Brandt’s voles (Lasiopodomys brandtii)
Source: Microbiome. 2022 Nov 15;10:194. doi: 10.1186/s40168-022-01381-1 (PMC9664686; doi:10.1186/s40168-022-01381-1)
Supplement: Supplementary file 6 — Additional file 5: Figure S5. Differences in diversity and composition of microbiota between the control groups and the FMT groups. [file 40168_2022_1381_MOESM5_ESM.docx]

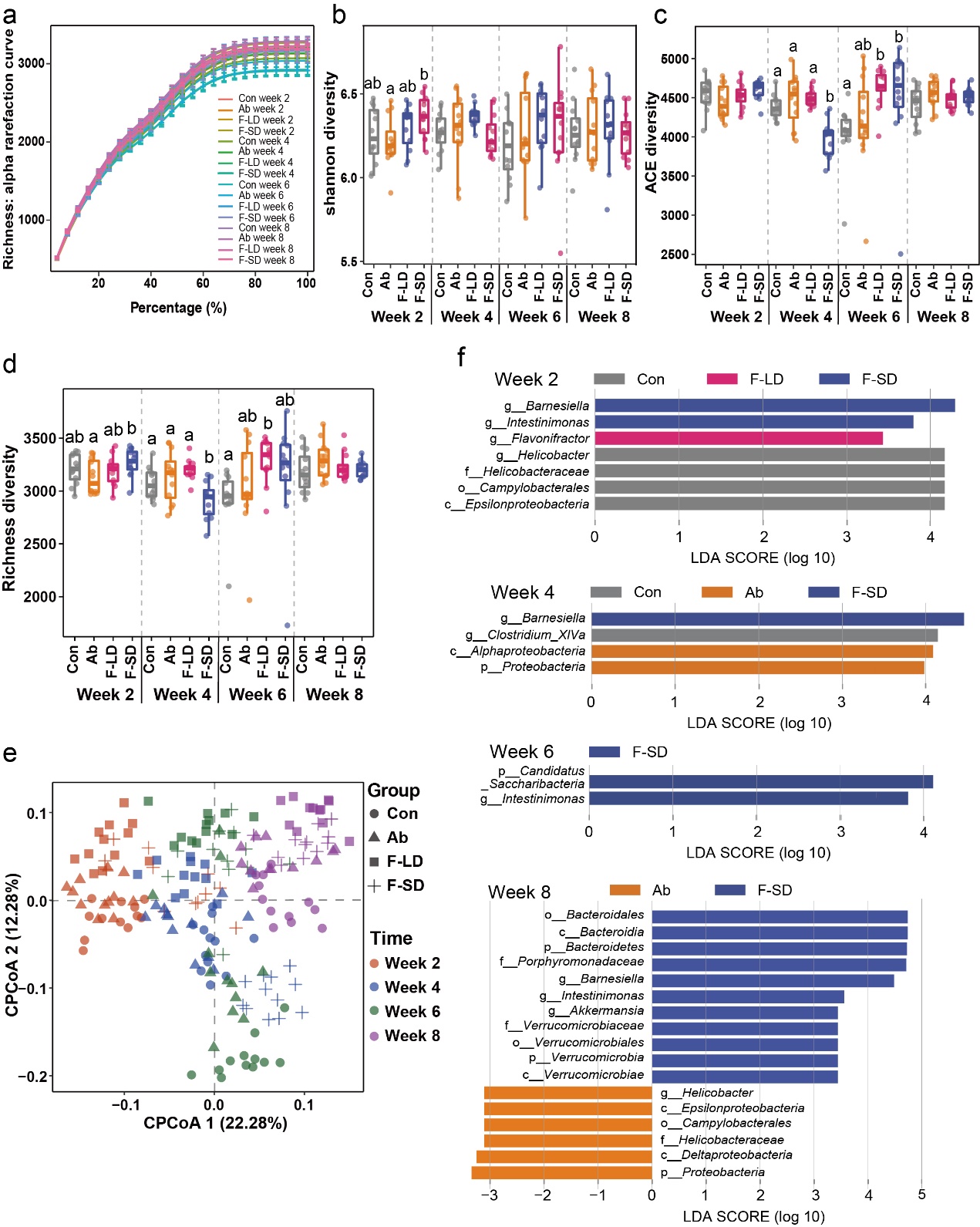


**Figure S5 Differences in diversity and composition of microbiota between the control groups and the FMT groups. a** Rarefaction curves of Richness diversity. **b-d** Shannon, ACE, and Richness indices (means ± SEM) of bacterial communities across the control and the FMT groups at week 2, 4, 6, and 8. **e** Constrained principal coordinate analysis (CPCoA) plot based on Bray-Curtis distance metrics shows the microbial community structure of samples from the control and the FMT groups. **f** The differentially abundant taxa enriched in microbial communities from the control and the FMT groups at week 2, 4, 6, and 8 by LEfSe (LDA >2, a < 0.05). Con: recipients with saline; Ab: recipients with antibiotic; F-LD: recipients with LD-exposed microbiota; F-SD: recipients with SD-exposed microbiota. Data are means ± SEM
