# Supplementary material for: Gut microbiota is associated with the effect of photoperiod on seasonal breeding in male Brandt’s voles (Lasiopodomys brandtii)
Source: Microbiome. 2022 Nov 15;10:194. doi: 10.1186/s40168-022-01381-1 (PMC9664686; doi:10.1186/s40168-022-01381-1)
Supplement: Supplementary file 7 — Additional file 6: Table S1. Information on gene primers of Brandt's voles. [file 40168_2022_1381_MOESM6_ESM.docx]

**Table S1 Information on gene primers of Brandt's vole**

| **Genes^a^** | **Accession No.** | **Sequence^b^ (5'-3')** | **Length** | **Annealing temperature** |
| --- | --- | --- | --- | --- |
| *Dio2* | KX856007 | F: GAAGAAGCACCGGAACCAAGAGG | 103 bp | 62°C |
|  |  | R: GTCCATGCGGTCAGCCACAAC |  |  |
| *Dio3* | KX889114 | F: CCACCCCGAGCCCGAAGTAG | 93 bp | 62°C |
|  |  | R: CACAGACGGTTGTCGCCTGATAC |  |  |
| *GnRH* | KY038929 | F: CGATTCTTTCCAAGAGATGGG | 124 bp | 62°C |
|  |  | R: CATCAGACTTTCCAGAGCTCCT |  |  |
| *Rfrp-3* | KY038930 | F: GACAAATATCTCCAGCCTAGAGG | 114 bp | 62°C |
|  |  | R: GGGCTGGACTCATCTTAATAACAT |  |  |
| *Kiss-1* | KX833248 | F: CACTGGCTTCTTGGCAGCTACTG | 143 bp | 62°C |
|  |  | R: GCCCTTTTCCCAGGCATTGA |  |  |
| *GPR54* | AF343726 | F: CTACTGTGCTGCGTGCCCTTC | 102 bp | 62°C |
|  |  | R: CGAGACCTGCTGGATGTAGTTGAC |  |  |
| *Stra8* | MK559416 | F: GCCAACAGCTTAGAGGAGGTCAAG | 130 bp | 62°C |
|  |  | R: CAACCTTCCCAACAGCCTCAGTG |  |  |
| *β-actin* | MK301451 | F: GCTCTCTTCCAGCCTTCCTTCCTG | 213 bp | 62°C |
|  |  | R: GTGTTGGCGTACAGGTCCTTGCGG |  |  |

^a^ *Dio2*: iodothyronine deiodinase 2; *Dio3*: iodothyronine deiodinase 3; *Kiss-1*: Kisspeptin-1; *GPR54*: G protein-coupled receptor 54; *GnRH*: encode gonadotropin-releasing hormone; *Rfrp-3*: RFamide-related peptide 3; *Stra8*: stimulated by retinoic acid 8. ^b^ F = forward; R = reverse.
