# Supplementary material for: Gut microbiota is associated with the effect of photoperiod on seasonal breeding in male Brandt’s voles (Lasiopodomys brandtii)
Source: Microbiome. 2022 Nov 15;10:194. doi: 10.1186/s40168-022-01381-1 (PMC9664686; doi:10.1186/s40168-022-01381-1)
Supplement: Supplementary file 8 — Additional file 7: Table S2. Spearman correlations between ASVs and serum hormones levels in the photoperiod experiment. [file 40168_2022_1381_MOESM7_ESM.docx]

**Table S2 Spearman correlations between ASVs and serum hormones levels in the photoperiod experiment.**

| **Genus** | **Hormones**  **ASVs** | **MT** | | **FSH** | | **LH** | | **GnRH** | | **T** | |
| --- | --- | --- | --- | --- | --- | --- | --- | --- | --- | --- | --- |
|  |  | ***r*** | ***P*** | ***r*** | ***P*** | ***r*** | ***P*** | ***r*** | ***P*** | ***r*** | ***P*** |
| *Barnesiella* | ASV_158 | **-0.548** | **0.006** | **0.546** | **0.006** | **0.746** | **0.000** | -0.077 | 0.719 | 0.201 | 0.345 |
|  | ASV_173 | 0.310 | 0.141 | -0.388 | 0.061 | -0.374 | 0.071 | 0.313 | 0.136 | -0.163 | 0.448 |
|  | ASV_197 | -0.481 | 0.017 | **0.539** | **0.007** | **0.720** | **0.000** | -0.043 | 0.842 | 0.290 | 0.168 |
|  | ASV_204 | -0.458 | 0.024 | 0.372 | 0.073 | **0.578** | **0.003** | 0.034 | 0.875 | 0.260 | 0.219 |
|  | ASV_263 | 0.495 | 0.014 | -0.482 | 0.017 | **-0.550** | **0.005** | 0.367 | 0.077 | -0.290 | 0.170 |
|  | ASV_48 | 0.315 | 0.134 | -0.319 | 0.129 | -0.332 | 0.113 | 0.244 | 0.250 | -0.117 | 0.585 |
|  | ASV_837 | -0.348 | 0.095 | **0.525** | **0.008** | 0.513 | 0.010 | -0.229 | 0.282 | 0.128 | 0.550 |
|  | ASV_20 | **-0.557** | **0.005** | 0.462 | 0.023 | 0.470 | 0.020 | -0.112 | 0.602 | 0.246 | 0.246 |
|  | ASV_596 | -0.501 | 0.013 | **0.658** | **0.000** | **0.704** | **0.000** | 0.103 | 0.633 | 0.125 | 0.561 |
| *Prevotella* | ASV_114 | **-0.536** | **0.007** | 0.491 | 0.015 | 0.492 | 0.015 | 0.137 | 0.525 | 0.159 | 0.459 |
|  | ASV_150 | **-0.515** | **0.010** | 0.460 | 0.024 | 0.388 | 0.061 | -0.344 | 0.100 | 0.348 | 0.096 |
|  | ASV_159 | **-0.662** | **0.000** | **0.566** | **0.004** | **0.579** | **0.003** | 0.112 | 0.603 | 0.197 | 0.356 |
|  | ASV_183 | -0.509 | 0.011 | **0.547** | **0.006** | **0.604** | **0.002** | -0.136 | 0.527 | 0.075 | 0.728 |
|  | ASV_1854 | **-0.544** | **0.006** | 0.483 | 0.017 | 0.460 | 0.024 | -0.010 | 0.961 | 0.302 | 0.152 |
|  | ASV_256 | **-0.527** | **0.008** | 0.317 | 0.131 | 0.298 | 0.157 | -0.144 | 0.503 | 0.336 | 0.108 |
|  | ASV_316 | **-0.549** | **0.005** | **0.546** | **0.006** | 0.427 | 0.037 | -0.349 | 0.094 | 0.482 | 0.017 |
| *Saccharibacteria_genera_incertae_sedis* | ASV_68 | **-0.623** | **0.001** | 0.430 | 0.036 | 0.367 | 0.078 | 0.058 | 0.787 | 0.066 | 0.759 |
| *Lactobacillus* | ASV_132 | **0.602** | **0.002** | -0.474 | 0.019 | **-0.549** | **0.005** | 0.069 | 0.747 | -0.115 | 0.591 |
| *Eubacterium* | ASV_270 | -0.510 | 0.011 | 0.422 | 0.040 | 0.390 | 0.060 | -0.426 | 0.038 | 0.155 | 0.470 |
| *Acetatifactor* | ASV_641 | **-0.580** | **0.003** | **0.563** | **0.004** | **0.606** | **0.002** | 0.023 | 0.916 | 0.158 | 0.460 |
| *Clostridium_XlVa* | ASV_120 | **0.586** | **0.003** | **-0.608** | **0.002** | **-0.559** | **0.005** | 0.074 | 0.731 | -0.306 | 0.146 |
|  | ASV_129 | **0.617** | **0.001** | **-0.632** | **0.001** | **-0.674** | **0.000** | 0.160 | 0.455 | -0.354 | 0.090 |
|  | ASV_143 | -0.429 | 0.036 | 0.458 | 0.024 | **0.519** | **0.009** | 0.275 | 0.193 | 0.499 | 0.013 |
|  | ASV_147 | 0.510 | 0.011 | **-0.558** | **0.005** | **-0.537** | **0.007** | 0.034 | 0.875 | -0.477 | 0.019 |
|  | ASV_161 | 0.431 | 0.036 | **-0.594** | **0.002** | **-0.583** | **0.003** | -0.020 | 0.926 | -0.389 | 0.060 |
|  | ASV_85 | -0.379 | 0.068 | 0.404 | 0.050 | **0.532** | **0.007** | -0.223 | 0.296 | 0.156 | 0.468 |
| *Roseburia* | ASV_520 | 0.251 | 0.237 | -0.346 | 0.098 | **-0.523** | **0.009** | 0.000 | 0.998 | -0.040 | 0.854 |
|  | ASV_678 | 0.347 | 0.096 | -0.362 | 0.082 | -0.391 | 0.059 | 0.077 | 0.722 | -0.130 | 0.546 |
| *Clostridium_IV* | ASV_70 | **-0.567** | **0.004** | 0.371 | 0.074 | 0.450 | 0.027 | -0.063 | 0.771 | 0.477 | 0.019 |
| *Flavonifractor* | ASV_738 | **-0.557** | **0.005** | 0.417 | 0.043 | 0.304 | 0.149 | -0.134 | 0.533 | 0.138 | 0.521 |
| *Ruminococcus* | ASV_17 | 0.283 | 0.179 | -0.424 | 0.039 | **-0.532** | **0.007** | -0.049 | 0.821 | -0.372 | 0.073 |
|  | ASV_683 | -0.397 | 0.055 | **0.574** | **0.003** | 0.482 | 0.017 | -0.184 | 0.391 | 0.120 | 0.575 |
|  | ASV_28 | 0.145 | 0.498 | -0.134 | 0.533 | -0.269 | 0.204 | 0.188 | 0.379 | 0.050 | 0.815 |

Correlation between gut microbiome (at ASVs levels) and serum hormones levels in long-day (LD) and short-day (SD) photoperiod after 8 weeks of photoperiod domestication. *r* and *P* represent correlation coefficient and significance between ASVs and serum hormones levels, respectively. Boldface indicates a significant correlation between ASVs and hormones (*|r|* > 0.5, *P* < 0.01). MT: melatonin; GnRH: gonadotropin-releasing hormone; FSH: follicle-stimulating hormone; LH: luteinizing hormone; T: testosterone.
