# Supplementary material for: Gut microbiota is associated with the effect of photoperiod on seasonal breeding in male Brandt’s voles (Lasiopodomys brandtii)
Source: Microbiome. 2022 Nov 15;10:194. doi: 10.1186/s40168-022-01381-1 (PMC9664686; doi:10.1186/s40168-022-01381-1)
Supplement: Supplementary file 9 — Additional file 8: Table S3. Spearman correlations between ASVs and hypothalamic genes in the photoperiod experiment. [file 40168_2022_1381_MOESM8_ESM.docx]

**Table S3 Spearman correlations between ASVs and hypothalamic genes in the photoperiod experiment.**

| **Genus** | **Genes**  **ASVs** | ***Dio2*** | | ***Kiss-1*** | | ***GPR54*** | | ***GnRH*** | | ***Rfrp-3*** | |
| --- | --- | --- | --- | --- | --- | --- | --- | --- | --- | --- | --- |
|  |  | ***r*** | ***P*** | ***r*** | ***P*** | ***r*** | ***P*** | ***r*** | ***P*** | ***r*** | ***P*** |
| *Barnesiella* | ASV_158 | 0.100 | 0.640 | **0.548** | **0.006** | -0.306 | 0.146 | -0.080 | 0.710 | 0.257 | 0.225 |
|  | ASV_173 | 0.186 | 0.384 | -0.307 | 0.145 | **0.518** | **0.010** | -0.104 | 0.627 | -0.238 | 0.262 |
|  | ASV_197 | 0.088 | 0.683 | 0.414 | 0.044 | -0.203 | 0.341 | -0.058 | 0.788 | 0.099 | 0.646 |
|  | ASV_204 | 0.104 | 0.629 | 0.357 | 0.087 | -0.272 | 0.198 | -0.147 | 0.494 | -0.008 | 0.971 |
|  | ASV_263 | 0.054 | 0.802 | -0.116 | 0.590 | 0.328 | 0.118 | -0.050 | 0.815 | -0.248 | 0.243 |
|  | ASV_48 | 0.187 | 0.382 | -0.257 | 0.226 | **0.529** | **0.008** | -0.153 | 0.475 | -0.300 | 0.154 |
|  | ASV_837 | -0.154 | 0.472 | 0.015 | 0.944 | -0.199 | 0.350 | 0.023 | 0.915 | 0.245 | 0.248 |
|  | ASV_20 | 0.102 | 0.636 | **0.523** | **0.009** | -0.363 | 0.081 | 0.223 | 0.294 | 0.025 | 0.907 |
|  | ASV_596 | 0.236 | 0.267 | **0.521** | **0.009** | -0.070 | 0.744 | -0.022 | 0.920 | 0.144 | 0.502 |
| *Prevotella* | ASV_114 | 0.177 | 0.408 | 0.126 | 0.557 | -0.324 | 0.122 | -0.109 | 0.613 | 0.140 | 0.513 |
|  | ASV_150 | -0.100 | 0.642 | 0.057 | 0.791 | -0.488 | 0.016 | -0.301 | 0.153 | 0.303 | 0.150 |
|  | ASV_159 | 0.187 | 0.381 | **0.523** | **0.009** | -0.094 | 0.661 | 0.078 | 0.716 | 0.286 | 0.175 |
|  | ASV_183 | -0.027 | 0.900 | 0.456 | 0.025 | -0.402 | 0.051 | -0.103 | 0.632 | 0.181 | 0.396 |
|  | ASV_1854 | 0.093 | 0.666 | 0.447 | 0.029 | -0.078 | 0.719 | 0.005 | 0.982 | 0.104 | 0.628 |
|  | ASV_256 | 0.054 | 0.802 | 0.259 | 0.222 | -0.460 | 0.024 | -0.026 | 0.903 | 0.015 | 0.945 |
|  | ASV_316 | -0.027 | 0.899 | 0.061 | 0.777 | -0.477 | 0.019 | -0.316 | 0.133 | 0.200 | 0.348 |
| *Saccharibacteria_genera_incertae_sedis* | ASV_68 | 0.201 | 0.347 | 0.186 | 0.384 | -0.179 | 0.402 | -0.161 | 0.453 | 0.282 | 0.182 |
| *Lactobacillus* | ASV_132 | -0.033 | 0.879 | -0.311 | 0.139 | 0.215 | 0.313 | -0.035 | 0.873 | -0.226 | 0.288 |
| *Eubacterium* | ASV_270 | -0.173 | 0.419 | 0.303 | 0.151 | **-0.647** | **0.001** | 0.080 | 0.710 | -0.028 | 0.897 |
| *Acetatifactor* | ASV_641 | 0.170 | 0.428 | 0.443 | 0.030 | -0.394 | 0.057 | 0.070 | 0.747 | 0.137 | 0.522 |
| *Clostridium_XlVa* | ASV_120 | -0.065 | 0.762 | -0.283 | 0.180 | **0.638** | **0.001** | -0.084 | 0.698 | -0.265 | 0.211 |
|  | ASV_129 | 0.037 | 0.865 | -0.277 | 0.191 | **0.527** | **0.008** | -0.017 | 0.939 | -0.372 | 0.073 |
|  | ASV_143 | 0.402 | 0.051 | 0.233 | 0.274 | -0.127 | 0.554 | -0.040 | 0.853 | 0.277 | 0.191 |
|  | ASV_147 | -0.037 | 0.865 | -0.339 | 0.105 | 0.298 | 0.157 | 0.264 | 0.213 | -0.182 | 0.394 |
|  | ASV_161 | -0.049 | 0.820 | -0.367 | 0.078 | 0.165 | 0.441 | 0.300 | 0.155 | -0.171 | 0.424 |
|  | ASV_85 | 0.002 | 0.994 | 0.258 | 0.223 | -0.246 | 0.246 | -0.025 | 0.907 | -0.045 | 0.834 |
| *Roseburia* | ASV_520 | -0.007 | 0.974 | -0.205 | 0.336 | -0.073 | 0.736 | 0.114 | 0.596 | -0.148 | 0.489 |
|  | ASV_678 | -0.144 | 0.501 | -0.389 | 0.060 | -0.009 | 0.968 | -0.103 | 0.632 | -0.157 | 0.464 |
| *Clostridium_IV* | ASV_70 | 0.166 | 0.438 | 0.270 | 0.201 | -0.473 | 0.020 | 0.027 | 0.900 | 0.175 | 0.414 |
| *Flavonifractor* | ASV_738 | -0.123 | 0.567 | 0.224 | 0.293 | -0.439 | 0.032 | -0.091 | 0.673 | 0.083 | 0.701 |
| *Ruminococcus* | ASV_17 | -0.020 | 0.926 | -0.054 | 0.802 | 0.328 | 0.118 | 0.094 | 0.662 | -0.032 | 0.881 |
|  | ASV_683 | -0.012 | 0.957 | 0.248 | 0.243 | -0.421 | 0.040 | -0.012 | 0.957 | 0.054 | 0.802 |
|  | ASV_28 | 0.070 | 0.744 | -0.451 | 0.027 | 0.004 | 0.984 | 0.035 | 0.872 | -0.203 | 0.340 |

Correlation between gut microbiome (at ASVs levels) and genes in the hypothalamus in long-day (LD) and short-day (SD) photoperiod after 8 weeks of photoperiod domestication. *r* and *P* represent correlation coefficient and significance between ASVs and genes in the hypothalamus, respectively. Boldface indicates a significant correlation between ASVs and genes in the hypothalamus (*|r|* > 0.5, *P* < 0.01). *Dio2:* iodothyronine deiodinase 2; *Kiss-1*: Kisspeptin-1; *GPR54*: G protein-coupled receptor 54; *GnRH*: encode gonadotropin-releasing hormone; *Rfrp-3*: RFamide-related peptide 3.
