# Supplementary material for: Gut microbiota is associated with the effect of photoperiod on seasonal breeding in male Brandt’s voles (Lasiopodomys brandtii)
Source: Microbiome. 2022 Nov 15;10:194. doi: 10.1186/s40168-022-01381-1 (PMC9664686; doi:10.1186/s40168-022-01381-1)
Supplement: Supplementary file 10 — Additional file 9: Table S4. Spearman correlation coefficients (r) between ASVs and testicular genes in the photoperiod experiment. [file 40168_2022_1381_MOESM9_ESM.docx]

**Table S4 Spearman correlation coefficients (*r*) between ASVs and testicular genes in the photoperiod experiment.**

| **Genus** | **Genes**  **ASVs** | ***Dio2*** | ***Dio3*** | ***Dio2/Dio3*** | ***Kiss-1*** | ***GPR54*** | ***GnRH*** | ***Stra8*** |
| --- | --- | --- | --- | --- | --- | --- | --- | --- |
|  |  | ***r*** | ***r*** | ***r*** | ***r*** | ***r*** | ***r*** | ***r*** |
| *Barnesiella* | ASV_158 | 0.273 | -0.396 | 0.428 | **0.631** | 0.355 | -0.278 | -0.394 |
|  | ASV_173 | -0.330 | 0.412 | -0.440 | -0.223 | -0.426 | -0.189 | 0.352 |
|  | ASV_197 | 0.229 | -0.306 | 0.343 | **0.530** | 0.307 | -0.354 | -0.299 |
|  | ASV_204 | 0.270 | -0.362 | 0.376 | 0.339 | 0.423 | 0.099 | -0.133 |
|  | ASV_263 | -0.272 | 0.359 | -0.305 | -0.104 | -0.359 | 0.274 | 0.327 |
|  | ASV_48 | -0.360 | 0.323 | -0.426 | -0.227 | -0.395 | -0.157 | 0.303 |
|  | ASV_837 | 0.194 | -0.378 | 0.306 | 0.251 | 0.207 | -0.289 | -0.309 |
|  | ASV_20 | 0.312 | -0.257 | 0.405 | 0.411 | 0.250 | -0.065 | -0.298 |
|  | ASV_596 | 0.157 | -0.496 | 0.405 | 0.455 | 0.372 | -0.298 | -0.415 |
| *Prevotella* | ASV_114 | 0.077 | -0.289 | 0.093 | 0.255 | 0.380 | -0.053 | -0.464 |
|  | ASV_150 | 0.182 | -0.074 | 0.092 | 0.103 | 0.381 | 0.004 | -0.154 |
|  | ASV_159 | 0.260 | -0.260 | 0.425 | **0.525** | 0.247 | -0.330 | -0.264 |
|  | ASV_183 | 0.138 | -0.474 | 0.359 | 0.479 | 0.369 | -0.008 | -0.407 |
|  | ASV_1854 | 0.178 | -0.083 | 0.278 | 0.439 | 0.159 | -0.307 | -0.078 |
|  | ASV_256 | 0.200 | -0.177 | 0.207 | 0.124 | 0.357 | 0.085 | -0.147 |
|  | ASV_316 | 0.170 | -0.189 | 0.128 | 0.090 | 0.440 | 0.010 | -0.229 |
| *Saccharibacteria_genera_incertae_sedis* | ASV_68 | -0.051 | -0.117 | 0.041 | 0.293 | 0.181 | -0.246 | -0.265 |
| *Lactobacillus* | ASV_132 | -0.356 | 0.511 | **-0.553** | -0.402 | -0.239 | 0.177 | 0.180 |
| *Eubacterium* | ASV_270 | 0.080 | -0.224 | 0.161 | 0.250 | 0.203 | -0.013 | -0.317 |
| *Acetatifactor* | ASV_641 | 0.121 | -0.239 | 0.220 | **0.517** | 0.298 | -0.307 | -0.425 |
| *Clostridium_XlVa* | ASV_120 | -0.267 | 0.239 | -0.251 | -0.401 | -0.453 | 0.123 | **0.525** |
|  | ASV_129 | -0.412 | 0.348 | -0.385 | -0.433 | -0.457 | 0.177 | **0.520** |
|  | ASV_143 | 0.190 | -0.008 | 0.136 | 0.256 | **0.516** | -0.226 | -0.293 |
|  | ASV_147 | -0.305 | 0.380 | -0.384 | -0.262 | **-0.517** | -0.019 | 0.190 |
|  | ASV_161 | -0.250 | 0.357 | -0.318 | -0.354 | -0.425 | 0.149 | 0.263 |
|  | ASV_85 | 0.196 | -0.471 | 0.355 | 0.274 | 0.257 | -0.100 | -0.256 |
| *Roseburia* | ASV_520 | -0.364 | 0.285 | -0.382 | -0.375 | -0.098 | 0.269 | 0.092 |
|  | ASV_678 | -0.329 | 0.370 | **-0.518** | -0.333 | -0.147 | 0.382 | 0.057 |
| *Clostridium_IV* | ASV_70 | 0.302 | -0.339 | 0.379 | 0.128 | **0.592** | 0.135 | -0.230 |
| *Flavonifractor* | ASV_738 | 0.167 | -0.421 | 0.269 | 0.171 | 0.236 | 0.278 | -0.271 |
| *Ruminococcus* | ASV_17 | -0.189 | 0.146 | -0.129 | -0.230 | -0.429 | 0.077 | 0.282 |
|  | ASV_683 | -0.085 | -0.234 | 0.062 | 0.208 | 0.245 | -0.211 | -0.402 |
|  | ASV_28 | -0.366 | 0.389 | **-0.528** | -0.408 | -0.162 | 0.037 | 0.036 |

Correlation between gut microbiome (at ASVs levels) and genes in testis in long-day (LD) and short-day (SD) photoperiod after 8 weeks of photoperiod domestication. *r* represents correlation coefficient between ASVs and genes in testis. Boldface indicates a significant correlation between ASVs and genes in testis (*|r|* > 0.5). *Dio2:* iodothyronine deiodinase 2; *Dio3*: iodothyronine deiodinase 3; *Dio2/Dio3*: the ratio of *Dio2* to *Dio3* expression; *Kiss-1*: Kisspeptin-1; *GPR54*: G protein-coupled receptor 54; *GnRH*: encode gonadotropin-releasing hormone; *Stra8*: stimulated by retinoic acid 8.
