# Supplementary material for: Gut microbiota is associated with the effect of photoperiod on seasonal breeding in male Brandt’s voles (Lasiopodomys brandtii)
Source: Microbiome. 2022 Nov 15;10:194. doi: 10.1186/s40168-022-01381-1 (PMC9664686; doi:10.1186/s40168-022-01381-1)
Supplement: Supplementary file 11 — Additional file 10: Table S5. Spearman correlations significance (P-value) between ASVs and testicular genes in the photoperiod experiment. [file 40168_2022_1381_MOESM10_ESM.docx]

**Table S5 Spearman correlations significance (*P*-value) between ASVs and testicular genes in the photoperiod experiment.**

| **Genus** | **Genes**  **ASVs** | ***Dio2*** | ***Dio3*** | ***Dio2/Dio3*** | ***Kiss-1*** | ***GPR54*** | ***GnRH*** | ***Stra8*** |
| --- | --- | --- | --- | --- | --- | --- | --- | --- |
|  |  | ***P*** | ***P*** | ***P*** | ***P*** | ***P*** | ***P*** | ***P*** |
| *Barnesiella* | ASV_158 | 0.197 | 0.055 | 0.037 | **0.001** | 0.088 | 0.189 | 0.057 |
|  | ASV_173 | 0.116 | 0.046 | 0.031 | 0.296 | 0.038 | 0.377 | 0.092 |
|  | ASV_197 | 0.282 | 0.146 | 0.101 | **0.008** | 0.145 | 0.090 | 0.156 |
|  | ASV_204 | 0.201 | 0.082 | 0.070 | 0.105 | 0.040 | 0.646 | 0.537 |
|  | ASV_263 | 0.199 | 0.085 | 0.147 | 0.630 | 0.085 | 0.195 | 0.119 |
|  | ASV_48 | 0.084 | 0.123 | 0.038 | 0.286 | 0.056 | 0.465 | 0.149 |
|  | ASV_837 | 0.363 | 0.068 | 0.146 | 0.237 | 0.332 | 0.171 | 0.141 |
|  | ASV_20 | 0.138 | 0.225 | 0.049 | 0.046 | 0.238 | 0.764 | 0.157 |
|  | ASV_596 | 0.465 | 0.014 | 0.049 | 0.026 | 0.074 | 0.157 | 0.044 |
| *Prevotella* | ASV_114 | 0.721 | 0.170 | 0.665 | 0.229 | 0.067 | 0.807 | 0.022 |
|  | ASV_150 | 0.395 | 0.730 | 0.668 | 0.632 | 0.066 | 0.985 | 0.474 |
|  | ASV_159 | 0.220 | 0.220 | 0.038 | **0.008** | 0.244 | 0.115 | 0.213 |
|  | ASV_183 | 0.520 | 0.019 | 0.085 | 0.018 | 0.076 | 0.969 | 0.049 |
|  | ASV_1854 | 0.405 | 0.699 | 0.188 | 0.032 | 0.457 | 0.145 | 0.719 |
|  | ASV_256 | 0.349 | 0.408 | 0.332 | 0.563 | 0.086 | 0.693 | 0.493 |
|  | ASV_316 | 0.426 | 0.378 | 0.551 | 0.677 | 0.032 | 0.961 | 0.281 |
| *Saccharibacteria_genera_incertae_sedis* | ASV_68 | 0.812 | 0.588 | 0.850 | 0.165 | 0.398 | 0.247 | 0.210 |
| *Lactobacillus* | ASV_132 | 0.088 | 0.011 | **0.005** | 0.052 | 0.260 | 0.408 | 0.401 |
| *Eubacterium* | ASV_270 | 0.710 | 0.292 | 0.453 | 0.240 | 0.342 | 0.950 | 0.131 |
| *Acetatifactor* | ASV_641 | 0.574 | 0.260 | 0.302 | **0.010** | 0.157 | 0.144 | 0.038 |
| *Clostridium_XlVa* | ASV_120 | 0.207 | 0.260 | 0.238 | 0.052 | 0.026 | 0.567 | **0.008** |
|  | ASV_129 | 0.045 | 0.096 | 0.063 | 0.035 | 0.025 | 0.407 | **0.009** |
|  | ASV_143 | 0.375 | 0.971 | 0.526 | 0.228 | **0.010** | 0.287 | 0.165 |
|  | ASV_147 | 0.147 | 0.067 | 0.064 | 0.216 | **0.010** | 0.929 | 0.373 |
|  | ASV_161 | 0.239 | 0.087 | 0.129 | 0.090 | 0.039 | 0.488 | 0.214 |
|  | ASV_85 | 0.360 | 0.020 | 0.089 | 0.195 | 0.225 | 0.643 | 0.228 |
| *Roseburia* | ASV_520 | 0.080 | 0.177 | 0.065 | 0.071 | 0.647 | 0.203 | 0.668 |
|  | ASV_678 | 0.116 | 0.075 | **0.010** | 0.112 | 0.492 | 0.066 | 0.793 |
| *Clostridium_IV* | ASV_70 | 0.152 | 0.105 | 0.068 | 0.552 | **0.002** | 0.530 | 0.281 |
| *Flavonifractor* | ASV_738 | 0.435 | 0.040 | 0.203 | 0.423 | 0.267 | 0.189 | 0.200 |
| *Ruminococcus* | ASV_17 | 0.377 | 0.496 | 0.549 | 0.281 | 0.037 | 0.722 | 0.182 |
|  | ASV_683 | 0.692 | 0.272 | 0.773 | 0.329 | 0.249 | 0.323 | 0.051 |
|  | ASV_28 | 0.079 | 0.060 | **0.008** | 0.048 | 0.450 | 0.865 | 0.869 |

Correlation between gut microbiome (at ASVs levels) and genes in testis in long-day (LD) and short-day (SD) photoperiod after 8 weeks of photoperiod domestication. *P* represent significance between ASVs and genes in testis. Boldface indicates a significant correlation between ASVs and genes in testis (*P* < 0.01). *Dio2:* iodothyronine deiodinase 2; *Dio3*: iodothyronine deiodinase 3; *Dio2/Dio3*: the ratio of *Dio2* to *Dio3* expression; *Kiss-1*: Kisspeptin-1; *GPR54*: G protein-coupled receptor 54; *GnRH*: encode gonadotropin-releasing hormone; *Stra8*: stimulated by retinoic acid 8.
