# Supplementary material for: Gut microbiota is associated with the effect of photoperiod on seasonal breeding in male Brandt’s voles (Lasiopodomys brandtii)
Source: Microbiome. 2022 Nov 15;10:194. doi: 10.1186/s40168-022-01381-1 (PMC9664686; doi:10.1186/s40168-022-01381-1)
Supplement: Supplementary file 12 — Additional file 11: Table S6. Spearman correlations between ASVs and serum hormones levels in the FMT experiment. [file 40168_2022_1381_MOESM11_ESM.docx]

**Table S6 Spearman correlations between ASVs and serum hormones levels in the FMT experiment.**

| **Genus** | **Hormones**  **ASVs** | **MT** | | **FSH** | | **LH** | | **GnRH** | | **T** | |
| --- | --- | --- | --- | --- | --- | --- | --- | --- | --- | --- | --- |
|  |  | ***r*** | ***P*** | ***r*** | ***P*** | ***r*** | ***P*** | ***r*** | ***P*** | ***r*** | ***P*** |
| *Barnesiella* | ASV_615 | 0.301 | 0.153 | -0.217 | 0.308 | -0.200 | 0.348 | -0.086 | 0.690 | -0.005 | 0.982 |
| *Prevotella* | ASV_1006 | **-0.596** | **0.002** | **0.635** | **0.001** | 0.426 | 0.038 | -0.039 | 0.856 | -0.032 | 0.882 |
| *Alistipes* | ASV_205 | 0.408 | 0.048 | -0.507 | 0.012 | **-0.544** | **0.006** | -0.108 | 0.614 | 0.040 | 0.854 |
| *Desulfovibrio* | ASV_514 | 0.198 | 0.353 | -0.123 | 0.565 | **-0.670** | **0.000** | -0.217 | 0.309 | 0.276 | 0.191 |
| *Saccharibacteria_genera_incertae_sedis* | ASV_171 | -0.181 | 0.398 | 0.485 | 0.016 | 0.239 | 0.260 | 0.013 | 0.953 | -0.237 | 0.264 |
|  | ASV_381 | -0.293 | 0.165 | 0.321 | 0.126 | 0.282 | 0.182 | 0.079 | 0.713 | 0.114 | 0.597 |
| *Clostridium_XlVa* | ASV_25 | -0.290 | 0.170 | 0.446 | 0.029 | 0.367 | 0.078 | 0.196 | 0.358 | -0.263 | 0.215 |
|  | ASV_256 | 0.276 | 0.191 | **-0.659** | **0.000** | -0.488 | 0.015 | -0.210 | 0.325 | 0.195 | 0.362 |
| *Roseburia* | ASV_99 | -0.378 | 0.068 | **0.604** | **0.002** | 0.061 | 0.778 | -0.031 | 0.885 | 0.056 | 0.796 |
| *Flavonifractor* | ASV_342 | -0.217 | 0.307 | **0.545** | **0.006** | 0.410 | 0.047 | 0.067 | 0.757 | -0.512 | 0.011 |
| *Oscillibacter* | ASV_484 | -0.336 | 0.108 | 0.416 | 0.043 | 0.120 | 0.576 | -0.125 | 0.560 | -0.037 | 0.864 |
| *Ruminococcus* | ASV_258 | **-0.606** | **0.002** | **0.697** | **0.000** | 0.172 | 0.421 | 0.041 | 0.849 | 0.021 | 0.924 |
|  | ASV_456 | -0.350 | 0.094 | **0.591** | **0.002** | 0.485 | 0.016 | 0.189 | 0.377 | 0.020 | 0.925 |
|  | ASV_324 | -0.079 | 0.715 | 0.268 | 0.205 | 0.401 | 0.052 | 0.123 | 0.565 | 0.083 | 0.699 |
|  | ASV_373 | -0.294 | 0.163 | 0.465 | 0.022 | 0.465 | 0.022 | 0.192 | 0.368 | -0.001 | 0.995 |

Correlation between gut microbiome (at ASVs levels) and serum hormones levels in F-LD and F-SD groups. *r* and *P* represent correlation coefficient and significance between ASVs and serum hormones levels, respectively. Boldface indicates a significant correlation between ASVs and hormones (*|r|* > 0.5, *P* < 0.01). MT: melatonin; GnRH: gonadotropin-releasing hormone; FSH: follicle-stimulating hormone; LH: luteinizing hormone; T: testosterone.
