# Supplementary material for: Gut microbiota is associated with the effect of photoperiod on seasonal breeding in male Brandt’s voles (Lasiopodomys brandtii)
Source: Microbiome. 2022 Nov 15;10:194. doi: 10.1186/s40168-022-01381-1 (PMC9664686; doi:10.1186/s40168-022-01381-1)
Supplement: Supplementary file 13 — Additional file 12: Table S7. Spearman correlations between ASVs and hypothalamic genes in the FMT experiment. [file 40168_2022_1381_MOESM12_ESM.docx]

**Table S7 Spearman correlations between ASVs and hypothalamic genes in the FMT experiment.**

| **Genus** | **Hormones**  **ASVs** | ***Dio2*** | | ***Kiss-1*** | | ***GPR54*** | | ***GnRH*** | | ***Rfrp-3*** | |
| --- | --- | --- | --- | --- | --- | --- | --- | --- | --- | --- | --- |
|  |  | ***r*** | ***P*** | ***r*** | ***P*** | ***r*** | ***P*** | ***r*** | ***P*** | ***r*** | ***P*** |
| *Barnesiella* | ASV_615 | 0.147 | 0.492 | -0.409 | 0.047 | **-0.557** | **0.005** | 0.308 | 0.143 | -0.102 | 0.635 |
| *Prevotella* | ASV_1006 | -0.246 | 0.246 | 0.342 | 0.102 | **0.621** | **0.001** | -0.395 | 0.056 | -0.020 | 0.924 |
| *Alistipes* | ASV_205 | 0.167 | 0.435 | -0.349 | 0.094 | -0.394 | 0.057 | 0.167 | 0.435 | -0.305 | 0.147 |
| *Desulfovibrio* | ASV_514 | 0.209 | 0.326 | -0.082 | 0.704 | -0.252 | 0.235 | -0.001 | 0.995 | -0.248 | 0.242 |
| *Saccharibacteria_genera_incertae_sedis* | ASV_171 | -0.502 | 0.012 | 0.179 | 0.402 | 0.260 | 0.220 | 0.078 | 0.716 | 0.143 | 0.506 |
|  | ASV_381 | -0.162 | 0.449 | 0.127 | 0.554 | 0.179 | 0.402 | 0.236 | 0.267 | 0.180 | 0.401 |
| *Clostridium_XlVa* | ASV_25 | 0.143 | 0.506 | **0.527** | **0.008** | 0.197 | 0.357 | -0.181 | 0.398 | 0.478 | 0.018 |
|  | ASV_256 | 0.371 | 0.074 | -0.318 | 0.130 | **-0.558** | **0.005** | 0.224 | 0.292 | 0.027 | 0.902 |
| *Roseburia* | ASV_99 | -0.255 | 0.230 | 0.304 | 0.148 | 0.397 | 0.055 | -0.239 | 0.260 | -0.210 | 0.324 |
| *Flavonifractor* | ASV_342 | -0.337 | 0.108 | 0.209 | 0.327 | 0.261 | 0.218 | -0.157 | 0.465 | 0.264 | 0.213 |
| *Oscillibacter* | ASV_484 | -0.342 | 0.101 | 0.147 | 0.493 | **0.586** | **0.003** | -0.031 | 0.886 | -0.107 | 0.619 |
| *Ruminococcus* | ASV_258 | -0.153 | 0.475 | 0.238 | 0.262 | **0.576** | **0.003** | -0.261 | 0.219 | -0.144 | 0.503 |
|  | ASV_456 | -0.411 | 0.046 | 0.091 | 0.672 | 0.458 | 0.025 | 0.102 | 0.635 | -0.057 | 0.790 |
|  | ASV_324 | **-0.518** | **0.010** | 0.172 | 0.422 | 0.175 | 0.414 | -0.243 | 0.252 | -0.216 | 0.311 |
|  | ASV_373 | **-0.518** | **0.009** | 0.028 | 0.898 | 0.501 | 0.013 | 0.183 | 0.391 | -0.071 | 0.742 |

Correlation between gut microbiome (at ASVs levels) and hypothalamic genes in F-LD and F-SD groups. *r* and *P* represent correlation coefficient and significance between ASVs and hypothalamic genes, respectively. Boldface indicates a significant correlation between ASVs and hypothalamic genes (*|r|* > 0.5, *P* < 0.01). *Dio2:* iodothyronine deiodinase 2; *Kiss-1*: Kisspeptin-1; *GPR54*: G protein-coupled receptor 54; *GnRH*: encode gonadotropin-releasing hormone; *Rfrp-3*: RFamide-related peptide 3.
