# Supplementary material for: Gut microbiota is associated with the effect of photoperiod on seasonal breeding in male Brandt’s voles (Lasiopodomys brandtii)
Source: Microbiome. 2022 Nov 15;10:194. doi: 10.1186/s40168-022-01381-1 (PMC9664686; doi:10.1186/s40168-022-01381-1)
Supplement: Supplementary file 14 — Additional file 13: Table S8. Spearman correlation coefficients (r) between ASVs and testicular genes in the FMT experiment. [file 40168_2022_1381_MOESM13_ESM.docx]

**Table S8 Spearman correlation coefficients (*r*) between ASVs and testicular genes in the FMT experiment.**

| **Genus** | **Genes**  **ASVs** | ***Dio2*** | ***Dio3*** | ***Dio2/Dio3*** | ***Kiss-1*** | ***GPR54*** | ***GnRH*** | ***Stra8*** |
| --- | --- | --- | --- | --- | --- | --- | --- | --- |
|  |  | ***r*** | ***r*** | ***r*** | ***r*** | ***r*** | ***r*** | ***r*** |
| *Barnesiella* | ASV_615 | 0.094 | 0.130 | -0.201 | 0.302 | -0.358 | -0.261 | **-0.619** |
| *Prevotella* | ASV_1006 | 0.004 | **-0.539** | **0.724** | 0.081 | -0.098 | 0.324 | 0.307 |
| *Alistipes* | ASV_205 | 0.114 | 0.189 | -0.221 | 0.475 | -0.111 | -0.117 | -0.362 |
| *Desulfovibrio* | ASV_514 | 0.417 | 0.315 | -0.126 | 0.340 | 0.123 | -0.214 | -0.012 |
| *Saccharibacteria_genera_incertae_sedis* | ASV_171 | -0.057 | -0.320 | 0.248 | **-0.629** | -0.033 | -0.022 | **0.550** |
|  | ASV_381 | -0.248 | **-0.534** | 0.414 | -0.116 | -0.363 | 0.187 | -0.024 |
| *Clostridium_XlVa* | ASV_25 | -0.385 | -0.115 | 0.064 | -0.212 | 0.057 | 0.052 | 0.182 |
|  | ASV_256 | -0.074 | 0.144 | -0.277 | 0.117 | -0.168 | 0.025 | **-0.569** |
| *Roseburia* | ASV_99 | 0.128 | -0.220 | 0.376 | -0.106 | 0.152 | 0.229 | 0.289 |
| *Flavonifractor* | ASV_342 | -0.070 | -0.171 | 0.251 | -0.036 | 0.194 | 0.123 | 0.381 |
| *Oscillibacter* | ASV_484 | 0.288 | -0.168 | 0.411 | 0.015 | 0.352 | 0.304 | 0.383 |
| *Ruminococcus* | ASV_258 | 0.158 | -0.331 | **0.522** | -0.047 | 0.003 | 0.239 | 0.206 |
|  | ASV_456 | 0.386 | -0.220 | 0.505 | 0.133 | 0.115 | 0.000 | 0.408 |
|  | ASV_324 | 0.147 | -0.196 | 0.288 | -0.174 | 0.006 | 0.171 | 0.443 |
|  | ASV_373 | 0.278 | -0.231 | 0.403 | -0.173 | 0.142 | -0.112 | 0.488 |

Correlation between gut microbiome (at ASVs levels) and testicular genes in F-LD and F-SD groups. *r* represents correlation coefficient between ASVs and testicular genes. Boldface indicates a significant correlation between ASVs and testicular genes (*|r|* > 0.5). *Dio2:* iodothyronine deiodinase 2; *Dio3*: iodothyronine deiodinase 3; *Dio2/Dio3*: the ratio of *Dio2* to *Dio3* expression; *Kiss-1*: Kisspeptin-1; *GPR54*: G protein-coupled receptor 54; *GnRH*: encode gonadotropin-releasing hormone; *Stra8*: stimulated by retinoic acid 8.
