# Supplementary material for: Gut microbiota is associated with the effect of photoperiod on seasonal breeding in male Brandt’s voles (Lasiopodomys brandtii)
Source: Microbiome. 2022 Nov 15;10:194. doi: 10.1186/s40168-022-01381-1 (PMC9664686; doi:10.1186/s40168-022-01381-1)
Supplement: Supplementary file 15 — Additional file 14: Table S9. Spearman correlations significance (P-value) between ASVs and testicular genes in the FMT experiment. [file 40168_2022_1381_MOESM14_ESM.docx]

**Table S9 Spearman correlation significance (*P*-value) between ASVs and testicular genes in the FMT experiment.**

| **Genus** | **Genes**  **ASVs** | ***Dio2*** | ***Dio3*** | ***Dio2/Dio3*** | ***Kiss-1*** | ***GPR54*** | ***GnRH*** | ***Stra8*** |
| --- | --- | --- | --- | --- | --- | --- | --- | --- |
|  |  | ***P*** | ***P*** | ***P*** | ***P*** | ***P*** | ***P*** | ***P*** |
| *Barnesiella* | ASV_615 | 0.661 | 0.546 | 0.345 | 0.152 | 0.086 | 0.218 | **0.001** |
| *Prevotella* | ASV_1006 | 0.986 | **0.007** | **0.000** | 0.707 | 0.650 | 0.123 | 0.145 |
| *Alistipes* | ASV_205 | 0.596 | 0.376 | 0.299 | 0.019 | 0.604 | 0.586 | 0.083 |
| *Desulfovibrio* | ASV_514 | 0.043 | 0.134 | 0.557 | 0.104 | 0.568 | 0.316 | 0.955 |
| *Saccharibacteria_genera_incertae_sedis* | ASV_171 | 0.790 | 0.127 | 0.243 | **0.001** | 0.878 | 0.920 | **0.005** |
|  | ASV_381 | 0.243 | **0.007** | 0.045 | 0.589 | 0.082 | 0.380 | 0.912 |
| *Clostridium_XlVa* | ASV_25 | 0.063 | 0.593 | 0.765 | 0.320 | 0.793 | 0.809 | 0.395 |
|  | ASV_256 | 0.730 | 0.502 | 0.190 | 0.587 | 0.434 | 0.908 | **0.004** |
| *Roseburia* | ASV_99 | 0.552 | 0.302 | 0.070 | 0.622 | 0.478 | 0.282 | 0.171 |
| *Flavonifractor* | ASV_342 | 0.747 | 0.426 | 0.236 | 0.869 | 0.365 | 0.567 | 0.066 |
| *Oscillibacter* | ASV_484 | 0.173 | 0.433 | 0.046 | 0.944 | 0.091 | 0.149 | 0.064 |
| *Ruminococcus* | ASV_258 | 0.462 | 0.115 | **0.009** | 0.829 | 0.990 | 0.261 | 0.333 |
|  | ASV_456 | 0.063 | 0.301 | 0.012 | 0.536 | 0.593 | 0.998 | 0.048 |
|  | ASV_324 | 0.492 | 0.359 | 0.172 | 0.417 | 0.976 | 0.424 | 0.030 |
|  | ASV_373 | 0.189 | 0.278 | 0.051 | 0.419 | 0.507 | 0.602 | 0.016 |

Correlation between gut microbiome (at ASVs levels) and testicular genes in F-LD and F-SD groups. *P* represent significance between ASVs and genes in testis*.* Boldface indicates a significant correlation between ASVs and testicular genes (*P* < 0.01). *Dio2:* iodothyronine deiodinase 2; *Dio3*: iodothyronine deiodinase 3; *Dio2/Dio3*: the ratio of *Dio2* to *Dio3* expression; *Kiss-1*: Kisspeptin-1; *GPR54*: G protein-coupled receptor 54; *GnRH*: encode gonadotropin-releasing hormone; *Stra8*: stimulated by retinoic acid 8.
