# Supplementary material for: Gut microbiota is associated with the effect of photoperiod on seasonal breeding in male Brandt’s voles (Lasiopodomys brandtii)
Source: Microbiome. 2022 Nov 15;10:194. doi: 10.1186/s40168-022-01381-1 (PMC9664686; doi:10.1186/s40168-022-01381-1)
Supplement: Supplementary file 16 — Additional file 15: Table S10. Spearman correlations of aggressive behavior with hormones and genes in the photoperiod experiment. [file 40168_2022_1381_MOESM15_ESM.docx]

**Table S10 Spearman correlations of aggressive behavior with hormones and genes in the photoperiod experiment.**

| **Physiological indicators** | | **LD** | | | | **SD** | | | |
| --- | --- | --- | --- | --- | --- | --- | --- | --- | --- |
|  |  | **Frequency of Attack** | | **Duration of Attack** | | **Frequency of Attack** | | **Duration of Attack** | |
|  |  | ***r*** | ***P*** | ***r*** | ***P*** | ***r*** | ***P*** | ***r*** | ***P*** |
| **Hormone** | **MT** | 0.244 | 0.445 | -0.042 | 0.896 | -0.071 | 0.827 | -0.014 | 0.965 |
|  | **FSH** | 0.380 | 0.223 | 0.113 | 0.726 | 0.127 | 0.694 | 0.099 | 0.760 |
|  | **LH** | 0.394 | 0.205 | 0.353 | 0.260 | -0.028 | 0.931 | 0.085 | 0.793 |
|  | **GnRH** | 0.201 | 0.532 | 0.353 | 0.260 | -0.297 | 0.349 | -0.226 | 0.480 |
|  | **T** | -0.294 | 0.354 | -0.396 | 0.203 | 0.170 | 0.598 | 0.240 | 0.452 |
| **Hypothalamus** | ***Dio2*** | 0.201 | 0.532 | 0.155 | 0.629 | -0.155 | 0.629 | -0.127 | 0.694 |
|  | ***Kiss-1*** | 0.308 | 0.330 | 0.254 | 0.425 | 0.198 | 0.538 | 0.240 | 0.452 |
|  | ***GPR54*** | 0.086 | 0.790 | -0.297 | 0.349 | -0.565 | 0.055 | -0.523 | 0.081 |
|  | ***GnRH*** | 0.108 | 0.739 | 0.028 | 0.931 | 0.191 | 0.552 | 0.333 | 0.291 |
|  | ***Rfrp-3*** | -0.029 | 0.929 | 0.382 | 0.221 | -0.396 | 0.203 | -0.424 | 0.169 |
| **Testis** | ***Dio2*** | 0.000 | 1.000 | 0.057 | 0.861 | 0.042 | 0.896 | 0.085 | 0.793 |
|  | ***Dio3*** | -0.022 | 0.947 | 0.424 | 0.169 | -0.028 | 0.931 | -0.155 | 0.629 |
|  | ***Dio2/Dio3*** | 0.022 | 0.947 | -0.269 | 0.399 | 0.085 | 0.793 | 0.212 | 0.508 |
|  | ***Kiss.1*** | 0.151 | 0.640 | 0.382 | 0.221 | -0.071 | 0.827 | -0.099 | 0.760 |
|  | ***GPR54*** | -0.158 | 0.624 | -0.226 | 0.480 | 0.297 | 0.349 | 0.325 | 0.302 |
|  | ***GnRH*** | -0.194 | 0.547 | 0.085 | 0.793 | **0.651** | **0.022** | **0.637** | **0.026** |
|  | ***Stra8*** | -0.258 | 0.418 | -0.353 | 0.260 | -0.325 | 0.302 | -0.396 | 0.203 |

MT: melatonin; GnRH: gonadotropin-releasing hormone; FSH: follicle-stimulating hormone; LH: luteinizing hormone; T: testosterone; *Dio2*: iodothyronine deiodinase 2; *Dio3*: iodothyronine deiodinase 3; *Dio2/Dio3*: the ratio of *Dio2* to *Dio3* expression; *Kiss*-1: Kisspeptin-1; *GPR54*: G protein-coupled receptor 54; *GnRH*: encode gonadotropin-releasing hormone; *Rfrp-3*: RFamide-related peptide 3; *Stra8*: stimulated by retinoic acid 8. LD: long-day photoperiod (16L: 8D); SD: short-day photoperiod (8L: 16D).
