# Supplementary material for: Gut microbiota is associated with the effect of photoperiod on seasonal breeding in male Brandt’s voles (Lasiopodomys brandtii)
Source: Microbiome. 2022 Nov 15;10:194. doi: 10.1186/s40168-022-01381-1 (PMC9664686; doi:10.1186/s40168-022-01381-1)
Supplement: Supplementary file 17 — Additional file 16: Table S11. Difference in physiological indices between the Con and Ab groups, Con and F-LD groups, Con and F-SD groups, Ab and F-LD groups, Ab and F-SD groups, and F-LD and F-SD groups in the FMT experiment. [file 40168_2022_1381_MOESM16_ESM.docx]

**Table S11 Difference in physiological indices between the Con and Ab groups, Con and F-LD groups, Con and F-SD groups, Ab and F-LD groups, Ab and F-SD groups, and F-LD and F-SD groups in the FMT experiment.**

| Physiological indicators | | Con vs Ab | | Con vs F-LD | | Con vs F-SD | | Ab vs F-LD | | Ab vs F-SD | | F-LD vs F-SD | |
| --- | --- | --- | --- | --- | --- | --- | --- | --- | --- | --- | --- | --- | --- |
|  |  | *t* | *P* | *t* | *P* | *t* | *P* | *t* | *P* | *t* | *P* | *t* | *P* |
| Hormone | MT | -0.512 | 0.614 | **-2.993** | **0.007** | **-7.063** | **0.000** | **-2.481** | **0.022** | **-6.551** | **0.000** | **-4.070** | **0.001** |
|  | FSH | **-4.464** | **0.000** | **3.026** | **0.007** | **5.224** | **0.000** | **7.490** | **0.000** | **9.688** | **0.000** | **2.197** | **0.040** |
|  | LH | 0.639 | 0.530 | **-8.612** | **0.000** | **-3.620** | **0.002** | **-9.251** | **0.000** | **-4.259** | **0.000** | **4.992** | **0.000** |
|  | GnRH | -0.676 | 0.507 | **-2.663** | **0.015** | **-2.122** | **0.047** | -1.987 | 0.061 | -1.446 | 0.164 | 0.541 | 0.595 |
|  | T | 1.316 | 0.203 | **-5.031** | **0.000** | **-3.919** | **0.001** | **-6.347** | **0.000** | **-5.236** | **0.000** | 1.112 | 0.279 |
| Hypothalamus | *Dio2* | -1.309 | 0.205 | 0.372 | 0.714 | -1.282 | 0.215 | 1.681 | 0.108 | 0.027 | 0.978 | -1.654 | 0.114 |
|  | *Kiss-1* | -0.345 | 0.734 | 0.258 | 0.799 | **2.489** | **0.022** | 0.603 | 0.553 | **2.835** | **0.010** | **2.231** | **0.037** |
|  | *GPR54* | **-3.503** | **0.002** | **-3.326** | **0.003** | -0.958 | 0.350 | 0.176 | 0.862 | **2.545** | **0.019** | **2.369** | **0.028** |
|  | *GnRH* | -0.614 | 0.546 | 0.224 | 0.825 | -0.288 | 0.776 | 0.838 | 0.412 | 0.326 | 0.748 | -0.512 | 0.614 |
|  | *Rfrp-3* | 0.301 | 0.767 | -1.493 | 0.151 | -0.583 | 0.566 | -1.794 | 0.088 | -0.884 | 0.387 | 0.909 | 0.374 |
| Testis | *Dio2* | **-4.067** | **0.001** | **-2.588** | **0.018** | **-2.185** | **0.041** | 1.478 | 0.155 | 1.881 | 0.075 | 0.403 | 0.691 |
|  | *Dio3* | -0.893 | 0.382 | 0.297 | 0.769 | **-2.225** | **0.038** | 1.191 | 0.248 | -1.331 | 0.198 | **-2.522** | **0.020** |
|  | *Dio2/Dio3* | **-2.204** | **0.039** | **-2.380** | **0.027** | 0.018 | 0.986 | -0.176 | 0.862 | **2.222** | **0.038** | **2.398** | **0.026** |
|  | *Kiss-1* | -0.605 | 0.552 | 0.945 | 0.356 | 0.759 | 0.457 | 1.549 | 0.137 | 1.363 | 0.188 | -0.186 | 0.854 |
|  | *GPR54* | -1.658 | 0.113 | -2.019 | 0.057 | -1.468 | 0.158 | -0.360 | 0.722 | 0.190 | 0.851 | 0.551 | 0.588 |
|  | *GnRH* | -0.683 | 0.503 | **-2.162** | **0.043** | -0.004 | 0.997 | -1.479 | 0.155 | 0.679 | 0.505 | **2.157** | **0.043** |
|  | *Stra8* | **-2.471** | **0.023** | **-3.251** | **0.004** | -2.008 | 0.058 | -0.781 | 0.444 | 0.463 | 0.649 | 1.243 | 0.228 |
| Genital organ | TWW | 0.309 | 0.761 | 0.564 | 0.579 | -0.510 | 0.616 | 0.255 | 0.801 | -0.819 | 0.422 | -1.074 | 0.296 |
|  | TWW/BM | 0.143 | 0.888 | 0.488 | 0.631 | -0.226 | 0.823 | 0.345 | 0.733 | -0.369 | 0.716 | -0.714 | 0.483 |
|  | EWW | 0.565 | 0.579 | -0.050 | 0.961 | -0.438 | 0.666 | -0.614 | 0.546 | -1.003 | 0.328 | -0.389 | 0.702 |
|  | EWW/BM | 0.524 | 0.606 | -0.236 | 0.816 | -0.005 | 0.996 | -0.760 | 0.456 | -0.528 | 0.603 | 0.231 | 0.819 |

MT: melatonin; GnRH: gonadotropin-releasing hormone; FSH: follicle-stimulating hormone; LH: luteinizing hormone; T: testosterone; *Dio2*: iodothyronine deiodinase 2; *Dio3*: iodothyronine deiodinase 3; *Dio2/Dio3*: the ratio of *Dio2* to *Dio3* expression; *Kiss*-1: Kisspeptin-1; *GPR54*: G protein-coupled receptor 54; *GnRH*: encode gonadotropin-releasing hormone; *Rfrp-3*: RFamide-related peptide 3; *Stra8*: stimulated by retinoic acid 8; TWW: testicular wet weight; TWW/BM: the ratio of testicular wet weight to body mass; EWW: epididymis wet weight; EWW/BM: the ratio of epididymis weight to body mass. Con: recipients with saline; Ab: recipients with antibiotic; F-LD: recipients with LD-exposed microbiota; F-SD: recipients with SD-exposed microbiota.
