# Supplementary material for: Gut microbiota is associated with the effect of photoperiod on seasonal breeding in male Brandt’s voles (Lasiopodomys brandtii)
Source: Microbiome. 2022 Nov 15;10:194. doi: 10.1186/s40168-022-01381-1 (PMC9664686; doi:10.1186/s40168-022-01381-1)
Supplement: Supplementary file 18 — Additional file 17: Table S12. Difference in physiological indices of gut microbiota between the Con and F-LD groups, Con and F-SD groups, Ab and F-LD groups, and Ab and F-SD groups in the FMT experiment. [file 40168_2022_1381_MOESM17_ESM.docx]

**Table S12 Difference in physiological indices of gut microbiota between the Con and F-LD groups, Con and F-SD groups, Ab and F-LD groups, and Ab and F-SD groups in the FMT experiment.**

| **Physiological indicators** | | **As compared with F-LD** | | **As compared with F-SD** | | **Four groups (LMM)** | |
| --- | --- | --- | --- | --- | --- | --- | --- |
|  |  | **Con** | **Ab** | **Con** | **Ab** | ***F*** | ***P*** |
| **Hormone** | **MT** | **🡓** | **🡓** | **🡓** | **🡓** | **20.789** | **0.000** |
|  | **FSH** | **🡑** | **🡑** | **🡑** | **🡑** | **35.192** | **0.000** |
|  | **LH** | **🡓** | **🡓** | **🡓** | **🡓** | **36.054** | **0.000** |
|  | **GnRH** | **🡓** | ns | **🡓** | ns | 3.063 | 0.052 |
|  | **T** | **🡓** | **🡓** | **🡓** | **🡓** | **18.558** | **0.000** |
| **Hypothalamus** | ***Dio2*** | ns | ns | ns | ns | 1.509 | 0.243 |
|  | ***Kiss-1*** | ns | ns | **🡑** | **🡑** | **3.294** | **0.042** |
|  | ***GPR54*** | **🡓** | ns | ns | **🡑** | **6.061** | **0.004** |
|  | ***GnRH*** | ns | ns | ns | ns | 0.263 | 0.851 |
|  | ***Rfrp-3*** | ns | ns | ns | ns | 1.248 | 0.319 |
| **Testis** | ***Dio2*** | **🡓** | ns | **🡓** | ns | **5.650** | **0.006** |
|  | ***Dio3*** | ns | ns | **🡓** | ns | 2.564 | 0.083 |
|  | ***Dio2/Dio3*** | **🡓** | ns | ns | **🡑** | **3.539** | **0.033** |
|  | ***Kiss-1*** | ns | ns | ns | ns | 1.021 | 0.404 |
|  | ***GPR54*** | ns | ns | ns | ns | 1.575 | 0.227 |
|  | ***GnRH*** | **🡓** | ns | ns | ns | 2.073 | 0.136 |
|  | ***Stra8*** | **🡓** | ns | ns | ns | **3.846** | **0.025** |
| **Genital organ** | **TWW** | ns | ns | ns | ns | 0.427 | 0.736 |
|  | **TWW/BM** | ns | ns | ns | ns | 0.179 | 0.909 |
|  | **EWW** | ns | ns | ns | ns | 0.341 | 0.796 |
|  | **EWW/BM** | ns | ns | ns | ns | 0.207 | 0.891 |

Upward/down arrow shows an increase/decrease in hormone levels or gene expression under Con or Ab groups as compared to F-LD and F-SD treatment. MT: melatonin; GnRH: gonadotropin-releasing hormone; FSH: follicle-stimulating hormone; LH: luteinizing hormone; T: testosterone; *Dio2*: iodothyronine deiodinase 2; *Dio3*: iodothyronine deiodinase 3; *Dio2/Dio3*: the ratio of *Dio2* to *Dio3* expression; *Kiss*-1: Kisspeptin-1; *GPR54*: G protein-coupled receptor 54; *GnRH*: encode gonadotropin-releasing hormone; *Rfrp-3*: RFamide-related peptide 3; *Stra8*: stimulated by retinoic acid 8; TWW: testicular wet weight; TWW/BM: the ratio of testicular wet weight to body mass; EWW: epididymis wet weight; EWW/BM: the ratio of epididymis weight to body mass. Con: recipients with saline; Ab: recipients with antibiotic; F-LD: recipients with LD-exposed microbiota; F-SD: recipients with SD-exposed microbiota.
