# Supplementary material for: Gut microbiota is associated with the effect of photoperiod on seasonal breeding in male Brandt’s voles (Lasiopodomys brandtii)
Source: Microbiome. 2022 Nov 15;10:194. doi: 10.1186/s40168-022-01381-1 (PMC9664686; doi:10.1186/s40168-022-01381-1)
Supplement: Supplementary file 19 — Additional file 18: Table S13. Difference in alpha diversity of gut microbiota between the Con and Ab groups, Con and F-LD groups, Con and F-SD groups, Ab and F-LD groups, Ab and F-SD groups, and F-LD and F-SD groups in the FMT experiment. [file 40168_2022_1381_MOESM18_ESM.docx]

**Table S13 Difference in alpha diversity of gut microbiota between the Con and Ab groups, Con and F-LD groups, Con and F-SD groups, Ab and F-LD groups, Ab and F-SD groups, and F-LD and F-SD groups in the FMT experiment.**

| **Alpha diversity** | **Week** | Con vs Ab | | Con vs F-LD | | Con vs F-SD | | Ab vs F-LD | | Ab vs F-SD | | F-LD vs F-SD | | All groups in all time (LMM) | |
| --- | --- | --- | --- | --- | --- | --- | --- | --- | --- | --- | --- | --- | --- | --- | --- |
|  |  | *t* | *P* | *t* | *P* | *t* | *P* | *t* | *P* | *t* | *P* | *t* | *P* | *F* | *P* |
| **Richness** | 2 | 1.155 | 0.3 | 0.203 | 0.8 | -0.961 | 0.3 | -0.952 | 0.4 | **-2.115** | **0.047** | -1.163 | 0.3 | Group: 3.20  Time: 4.33 | Group: 0.03  Time: 0.01 |
|  | 4 | -0.842 | 0.4 | -1.804 | 0.1 | **2.151** | **0.04** | -0.962 | 0.3 | **2.993** | **0.007** | **3.955** | **0.001** |  |  |
|  | 6 | -0.737 | 0.5 | **-2.340** | **0.03** | -1.537 | 0.1 | -1.603 | 0.1 | -0.800 | 0.4 | 0.803 | 0.4 |  |  |
|  | 8 | -1.282 | 0.2 | -0.575 | 0.6 | -0.269 | 0.8 | 0.708 | 0.5 | 1.014 | 0.3 | 0.306 | 0.8 |  |  |
| **Shannon** | 2 | 0.581 | 0.6 | -0.783 | 0.4 | -1.802 | 0.1 | -1.364 | 0.2 | **-2.382** | **0.027** | -1.018 | 0.3 | Group: 1.88  Time: 0.13 | Group: 0.2  Time: 0.9 |
|  | 4 | -0.323 | 0.8 | -1.729 | 0.1 | 0.310 | 0.8 | -1.406 | 0.2 | 0.632 | 0.5 | 2.038 | 0.055 |  |  |
|  | 6 | -0.744 | 0.5 | -1.568 | 0.1 | -1.070 | 0.3 | -0.824 | 0.4 | -0.326 | 0.7 | 0.498 | 0.6 |  |  |
|  | 8 | -0.277 | 0.8 | -0.314 | 0.8 | 0.073 | 0.9 | -0.038 | 0.97 | 0.350 | 0.7 | 0.387 | 0.7 |  |  |
| **ACE** | 2 | 1.359 | 0.2 | 0.308 | 0.8 | -0.701 | 0.5 | -1.051 | 0.3 | -2.060 | 0.053 | -1.009 | 0.3 | Group: 2.97  Time: 5.22 | Group:0.047  Time: 0.004 |
|  | 4 | -1.022 | 0.3 | -1.260 | 0.2 | **4.292** | **0.000** | -0.237 | 0.8 | **5.314** | **0.000** | **5.551** | **0.000** |  |  |
|  | 6 | -0.697 | 0.5 | **-2.685** | **0.01** | **-2.144** | **0.045** | -1.988 | 0.06 | -1.447 | 0.1 | 0.541 | 0.6 |  |  |
|  | 8 | -1.504 | 0.1 | -0.756 | 0.5 | -1.057 | 0.3 | 0.749 | 0.5 | 0.448 | 0.7 | -0.301 | 0.8 |  |  |

Con: recipients with saline; Ab: recipients with antibiotic; F-LD: recipients with LD-exposed microbiota; F-SD: recipients with SD-exposed microbiota.
