# Supplementary material for: Gut microbiota is associated with the effect of photoperiod on seasonal breeding in male Brandt’s voles (Lasiopodomys brandtii)
Source: Microbiome. 2022 Nov 15;10:194. doi: 10.1186/s40168-022-01381-1 (PMC9664686; doi:10.1186/s40168-022-01381-1)
Supplement: Supplementary file 20 — Additional file 19: Table S14. Difference in beta diversity of gut microbiota between the Con and Ab groups, Con and F-LD groups, Con and F-SD groups, Ab and F-LD groups, Ab and F-SD groups, and F-LD and F-SD groups in the FMT experiment. [file 40168_2022_1381_MOESM19_ESM.docx]

**Table S14 Difference in beta diversity of gut microbiota between the Con and Ab groups, Con and F-LD groups, Con and F-SD groups, Ab and F-LD groups, Ab and F-SD groups, and F-LD and F-SD groups in the FMT experiment.**

| **Time** | **Con vs Ab** | | **Con vs F-LD** | | **Con vs F-SD** | | **Ab vs F-LD** | | **Ab vs F-SD** | | **F-LD vs F-SD** | |
| --- | --- | --- | --- | --- | --- | --- | --- | --- | --- | --- | --- | --- |
|  | ***F*** | ***P*** | ***F*** | ***P*** | ***F*** | ***P*** | ***F*** | ***P*** | ***F*** | ***P*** | ***F*** | ***P*** |
| **Week 2** | 1.22 | 0.113 | **1.96** | **0.000** | **2.27** | **0.000** | **1.35** | **0.043** | **2.28** | **0.000** | **2.04** | **0.000** |
| **Week 4** | 1.15 | 0.200 | **1.94** | **0.000** | **3.11** | **0.000** | 1.14 | 0.207 | **3.00** | **0.000** | **3.18** | **0.000** |
| **Week 6** | **1.65** | **0.000** | **3.61** | **0.000** | **3.19** | **0.000** | **2.19** | **0.000** | **2.26** | **0.000** | **1.70** | **0.003** |
| **Week 8** | **1.54** | **0.008** | **2.07** | **0.000** | **2.10** | **0.000** | **1.70** | **0.003** | **2.01** | **0.000** | **1.74** | **0.000** |

Con: recipients with saline; Ab: recipients with antibiotic; F-LD: recipients with LD-exposed microbiota; F-SD: recipients with SD-exposed microbiota. PERMANOVA (adonis function in R, permutation = 9999)
